# Hairy Canola (*Brasssica napus*) re-visited: Down-regulating *TTG1* in an *AtGL3*-enhanced hairy leaf background improves growth, leaf trichome coverage, and metabolite gene expression diversity

**DOI:** 10.1186/s12870-015-0680-5

**Published:** 2016-01-06

**Authors:** Ushan I. Alahakoon, Ali Taheri, Naghabushana K. Nayidu, Delwin Epp, Min Yu, Isobel Parkin, Dwayne Hegedus, Peta Bonham-Smith, Margaret Y. Gruber

**Affiliations:** Saskatoon Research Centre, Agriculture and Agri-Food Canada, 107 Science Place, Saskatoon, SK S7N0X2 Canada; Department of Biology, University of Saskatchewan, 112 Science Place, Saskatoon, SK S7N5E2 Canada; Present address: DOW Agro-Sciences, 101-421 Downey Rd., Saskatoon, SK S7N4L8 Canada; Present address: Department of Agriculture and Environmental Sciences, Tennessee State University, 3500 John A Merritt Blvd., Nashville, TN 37209 USA

**Keywords:** *Brassica napus*, *GL3* and *TTG1* manipulation, Trichome patterning and growth, Broad metabolic gene expression changes, Q-PCR and RNA sequencing

## Abstract

**Background:**

Through evolution, some plants have developed natural resistance to insects by having hairs (trichomes) on leaves and other tissues. The hairy trait has been neglected in Brassica breeding programs, which mainly focus on disease resistance, yield, and overall crop productivity. In Arabidopsis, a network of three classes of proteins consisting of TTG1 (a WD40 repeat protein), GL3 (a bHLH factor) and GL1 (a MYB transcription factor), activates trichome initiation and patterning. Introduction of a trichome regulatory gene *AtGL3* from Arabidopsis into semi-glabrous *Brassica napus* resulted in hairy canola plants which showed tolerance to flea beetles and diamondback moths; however plant growth was negatively affected. In addition, the role of *BnTTG1* transcription in the new germplasm was not understood.

**Results:**

Here, we show that two ultra-hairy lines (K-5-8 and K-6-3) with *BnTTG1* knock-down in the hairy AtGL3+ *B. napus* background showed stable enhancement of trichome coverage, density, and length and restored wild type growth similar to growth of the semi-glabrous Westar plant. In contrast, over-expression of *BnTTG1* in the hairy AtGL3+ *B. napus* background gave consistently glabrous plants of very low fertility and poor stability, with only one glabrous plant (O-3-7) surviving to the T_3_ generation. Q-PCR trichome gene expression data in leaf samples combining several leaf stages for these lines suggested that *BnGL2* controlled *B. napus* trichome length and out-growth and that strong *BnTTG1* transcription together with strong *GL3* expression inhibited this process. Weak expression of *BnTRY* in both glabrous and trichome-bearing leaves of *B. napus* in the latter Q-PCR experiment suggested that *TRY* may have functions other than as an inhibitor of trichome initiation in the Brassicas. A role for *BnTTG1* in the lateral inhibition of trichome formation in neighbouring cells was also proposed for *B. napus*. RNA sequencing of first leaves identified a much larger array of genes with altered expression patterns in the K-5-8 line compared to the hairy AtGL3^+^*B. napus* background (relative to the Westar control plant). These genes particularly included transcription factors, protein degradation and modification genes, but also included pathways that coded for anthocyanins, flavonols, terpenes, glucosinolates, alkaloids, shikimates, cell wall biosynthesis, and hormones. A 2nd Q-PCR experiment was conducted on redox, cell wall carbohydrate, lignin, and trichome genes using young first leaves, including T_4_ O-3-7-5 plants that had partially reverted to yield two linked growth and trichome phenotypes. Most of the trichome genes tested showed to be consistant with leaf trichome phenotypes and with RNA sequencing data in three of the lines. Two redox genes showed highest overall expression in K-5-8 leaves and lowest in O-3-7-5 leaves, while one redox gene and three cell wall genes were consistently higher in the two less robust lines compared with the two robust lines.

**Conclusion:**

The data support the strong impact of *BnTTG1* knockdown (in the presence of strong *AtGL3* expression) at restoring growth, enhancing trichome coverage and length, and enhancing expression and diversity of growth, metabolic, and anti-oxidant genes important for stress tolerance and plant health in *B. napus*. Our data also suggests that the combination of strong (up-regulated) *BnTTG1* expression in concert with strong At*GL3* expression is unstable and lethal to the plant.

**Electronic supplementary material:**

The online version of this article (doi:10.1186/s12870-015-0680-5) contains supplementary material, which is available to authorized users.

## Background

Trichomes are epidermal hairs that serve as a physical barrier on plant surfaces against insect pests, while maintaining a moist microclimate around young seedlings. Defence against insect pests depends on trichome density, distribution pattern, length, shape, number of branches, and on whether they are glandular or non-glandular. Non-glandular trichome development has been studied extensively in Arabidopsis [1–3] a close relative of *B. napus* [4, 5]. A minimum of 21 genome blocks are conserved, but replicated and rearranged in the present day *B. napus* genome compared with the Arabidopsis genome [5], such that an estimated two to six homologues per gene are expected in *B. napus* [6]. Many differences exist between these two long-diverged genera, one being that the Brassicas have unbranched trichomes (Taheri and Nayidu, unpublished) whereas Arabidopsis trichomes are mostly tri-branched [7, 8].

A range of mutants defining specific aspects of trichome development have been recovered from Arabidopsis [8], and >70 AGI models are commonly known [listed in 2 and 61] or have been listed in The Arabidopsis Information Resource (Table S3 in [8]). *TRANSPARENT TESTA GLABRA1* (*TTG1*) [9], *GLABRA3* (*GL3*) [10], *ENHANCER OF GLABRA3* (*EGL3*) [10], *GLABROUS1* (*GL1*) [11], and to some extent *MYB23* [12], are genes specifying proteins involved in Arabidopsis trichome initiation. *GL3* is moderately expressed in epidermal cells of young leaves prior to trichome initiation but is highly expressed in initiating trichome cells, becoming less detectable or non-existent in mature trichomes [10]. GL3 (a bHLH protein) functions with GL1 (MYB) and TTG1 (WD40) to form a MBW activator complex. Over-expression of a *35S:GL3* cDNA in wild type Arabidopsis results in an increased trichome number phenotype [13], while over-expression of *GL1* and *GL3* together stimulated a “super-dense” leaf trichome phenotype (20 to 30-fold greater than wild type plants), with a few trichomes additionally present on cotyledons and hypocotyls [13]. The pleiotropic *TTG1* locus regulates trichome initiation, as well as flavonoid production, seed coat mucilage production, and root hair development pathways in Arabidopsis [9] and its transcripts are present to some extent in almost all organs of wild type Arabidopsis [9]. Arabidopsis *ttg1* mutants are not able to initiate trichomes [14]; however, clusters of rudimentary trichomes are formed along the leaf margin in weak *ttg1* mutants suggesting an additional contrasting role in trichome repression for TTG1 that has yet to be explored [15]. Mutations in *LEAFY COTYLEDON1* (*LEC1*) result in trichomes on the adaxial surface of the cotyledons, where trichomes are not normally produced [16].

The WRKY transcription factor, TTG2, initially acts downstream of TTG1, (E)GL3, and GL1, and contributes with other proteins (such as SAD2) in trichome initiation [17, 18]. Later in trichome development, TTG2 and GL2 regulate lateral out-growth, morphological development, and maturation of trichomes [18, 19]. GL2 is normally expressed in developing trichomes and surrounding epidermal cells of leaf primordia, but is also detected in the petiole and mid-vein of developing leaves. Increased trichome size correlates with increased GL2 activity, which remains high in mature trichomes for the life time of the cell [19]. In both Arabidopsis *gl1* and *ttg1* single mutants, *GL2* expression is undetectable in epidermal cells of fully expanded mature leaves, suggesting that *GL2* expression is regulated by both GL1 and TTG1 [19].

In the activator-inhibitor model of Arabidopsis trichome initiation, the MBW tri-protein complex or di-protein GL1-GL3/EGL3 complexes not only promote GL2 expression to activate trichome formation [2, 20], but also initiate transcription of inhibitor genes. All seven of the single-repeat R3MYB MYB inhibitor proteins (lacking the R2 activator domain) can physically interact with GL3 and EGL3 [1] as well as compete with GL1 [21] to interact with these latter bHLH proteins. Although R3MYB competition limits the transcriptional activity of the MBW complex, epistatic analysis of *gl2* and single *myb* Arabidopsis mutants suggest that the R3MYB inhibitors do not act directly through GL2 in regulating trichome formation [22]. TTG2 shares function with GL2 in the trichome development cascade and its expression is also controlled by the MBW complex [23].

The seven R3MYB inhibitor transcription factors inhibiting trichome development include *TRIPTYCHON* (*TRY*) [24], *CAPRICE* (*CPC*) [25], *ENHANCER OF TRY AND CPC 1* (*ETC1*) [26], *ETC2*(or *3*) [26], *CAPRICE-LIKE MYB 3* (*CPL3*) [27], *TRICHOMELESS1* (*TCL1*) [28], and *TCL2* [29]. *REDUCED TRICHOME NUMBER* (*RTN*) affects the number of trichomes produced per leaf, and consequently increases the distance between trichomes on mature leaves [30]. Over-expression of any of the seven R3MYB negative regulatory genes results in a glabrous phenotype in Arabidopsis, while mutants of each of these genes show different phenotypes. Root hair development is also affected by these genes.

While extensive molecular knowledge exists for trichome development in Arabidopsis, an equivalent knowledge base is lacking for Brassica crop species. Complementation of a glabrous, yellow-seeded Arabidopsis *ttg1* mutant with a *BrTTG1* ortholog isolated from a hairy, black-seeded *B. rapa* line restores brown seed colour, trichome density, seed coat mucilage, anthocyanins, and reduces root hair density to the wild type level, suggesting that *BnTTG1* functions in a similar manner to the Arabidopsis *AtTTG1* [31]. Five of the best known trichome regulatory genes have been recently studied in *B. villosa* [32, 33], which has an extremely dense coverage of trichomes over most of its tissues [34]. Transformation of *B. napus* with *35S*_*p*_*::AtGL3* produces an extremely dense covering of trichomes on *B. napus* seedling tissues [35]. This “Hairy Canola” line (called AtGL3^+^*B. napus*) shows strong feeding deterrence to crucifer flea beetles [36] and modest resistance to larvae of the diamondback moth [37]. However, it also showed stunted growth, and after five generations these plants were still more than one week late in maturation. Moreover, the impact of *TTG1* transcript levels relative to those of other trichome regulatory genes in the Brassicas and the molecular basis of trichome development in most Brassica species are still unknown [38], although genome sequencing is now offering exciting new opportunities to study these genes [39, 40]. Additionally, the effects that trichome development genes may have on Brassica metabolite pathways, overall plant development, growth, and seed yield have yet to be evaluated.

In an attempt to address several of these issues, *BnTTG1* transcript levels were modified in two *B. napus* recipient genetic backgrounds: semi-glabrous cv Westar and the “Hairy Canola” line AtGL3^+^*B. napus*. The reduction of *BnTTG1* transcript levels in the hairy AtGL3^+^*B. napus* background resulted in new ultra-hairy “Hairy Canola” lines, with high trichome density, expanded trichome coverage and length, and restored growth and time to maturity. In contrast, over-expression of *BnTTG1* in the hairy background resulted in loss of vitality and a glabrous (but unstable) trichome and growth phenotype. Transcriptome analyses in young leaves of one of the ultra-hairy lines and the original hairy line provide insight into the impact of the combined manipulation of *AtGL3* and *BnTTG1* gene expression on expression of various trichome, plant growth, biochemical, and defence pathways in *B. napus*.

## Results

### Transgenic plant growth and fertility phenotypes

None of the *BnTTG1* over-expression or knockdown lines in the semi-glabrous leaf Westar recipient background showed any phenotypic differences with respect to plant morphology, trichome coverage, growth or reproduction when compared to Westar. Hence, representatives of these plants were not advanced to produce homozygous lines due to greenhouse space limitations.

**Fig. 1:**
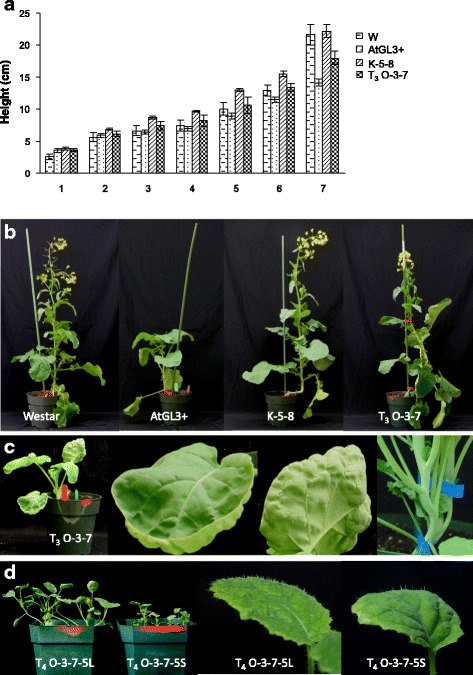
Greenhouse growth and plant phenotypes for semi-glabrous *B. napus* cv Westar (W), hairy AtGL3^+^ *B. napus* (AtGL3^+^), ultra-hairy K-5-8 (*BnTTG1* knock-down in AtGL3^+^ *B. napus*), and glabrous or moderately hairy O-3-7 (*BnTTG1* over-expression in AtGL3^+^ *B. napus*). **a** Plant height over a 7 week period with glabrous T_3_ O-3-7 plants (size bar represents 1 cm). **b** Growth phenotypes (with glabrous T_3_ O-3-7) at 8 weeks. Means (+ SE) of six plants per line with different letters are significantly different at *P* ≤ 0.05. C,D. Unstable growth and trichome phenotypes in O-3-7 plants. **c** T_3_ O-3-7 seedlings growing in panel B, showing glabrous leaves (left adaxial; right abaxial) and glabrous stems. **d**. T_4_ O-3-7 revertants with two different phenotypes (larger/healthy plants with moderately hairy leaves and small/unhealthy with poor viability and leaf hair density somewhat higher than Westar)

In contrast, the O-TTG1 lines (with over-expression of *BnTTG1* within the hairy leaf AtGL3^+^*B. napus* recipient background) showed substantial changes to phenotype and were usually non-viable (Table 1). Of the seven confirmed O-TTG1 T_0_ transformants in the hairy background, five showed slow growth, a fully glabrous phenotype, and did not flower, eventually dying. Only two single-insertion T_0_ glabrous O-TTG1 plants (O-3 and O-9) survived to flowering after transfer to soil. Both of these plants grew taller than the AtGL3^+^*B. napus* plants, but only O-3 produced siliques (resulting in a total of five seeds, most of which were mishapen). Of the five T_1_ O-3 seeds, only one germinated and produced a plant with seeds. Of ten glabrous progeny T_2_ seeds, only three germinated and only one of the resulting glabrous plants (O-3-7) flowered and produced seeds. Leaves of all T_3_ O-3-7 plants were dark green and glabrous, and the plants were taller and faster growing compared to AtGL3^+^*B. napus* plants and T_1_ O-3 seed, but slower and with shorter stature when evaluated side-by-side with Westar for growth (Fig. 1a, b). These somewhat healthier glabrous O-3-7 seedlings (Fig. 1c) also produced 20 % less seed of lower quality (cracked or oddly shaped) under greenhouse conditions (as did hairy AtGL3^+^*B. napus*) compared to Westar.

**Table 1:** Transformation efficiency with *BnTTG1* manipulation: Survival and viable seed set of confirmed transformants

Genetic background	Binary construct	No. of explants	Putative T_0_ PPT-tolerant plantlets^a^	Confirmed T_0_transgenic plants^a^	Single insertion locus plants^a^	T_0_ plants with viable seed set
*B. napus* Westar	O-TTG1	500	20	12	5	10
	K-TTG1	500	20	7	2	5
AtGL3^+^ *B. napus*	O-TTG1	500	9	7	2	1
	K-TTG1	500	10	9	4	5

^a^Transgene presence was confirmed by initial screening using phosphinothricin selection, followed by PCR (Additional file 1: Figure S2) and Southern blotting (Additional file 1: Figure S3) of the selectable marker gene (*BAR*) to confirm transgene loci number

Sixteen months later, T_4_ O-3-7 seedlings from two different seed lots (O-3-7-3 and O-3-7-5) were each grown over two periods (and both in soil and in Magenta boxes) to re-evaluate the plants prior to 1st leaf Q-PCR analysis. Each seed lot, growth period, and growth condition generated the same two unique growth phenotypes in a 1:1 segregation ratio, both of which included only trichome-bearing leaves. Phenotype 1 plants grew moderately tall like the AtGL3^+^*B. napus* line and T_3_ O-3-7 plants, while phenotype 2 grew only to ~2 cm in height (1/4 to 1/2 the height phenotype 1), then became yellowish and died (Fig. 1d).

Of the nine independent K-TTG1 positive lines in AtGL3^+^*B. napus*, four T_0_ plants contained a single transgene insert and presented two distinct growth and trichome phenotypes. K8 and K10 plants showed growth characteristics and leaf/stem trichome coverage similar to AtGL3^+^*B. napus* plants; however, K-5 and K6 plants showed restored growth characteristics identical to Westar and new trichome phenotypes detailed below. From these latter plants, the T_3_ homozygous lines K-5-8 and K-6-3 were developed. In greenhouse growth studies, K-6-3 and K-5-8 grew more vigorously than AtGL3^+^*B. napus* plants and O-3-7 plants resulting in robust plants similar to Westar (Fig. 1a, b; shown only for K-5-8 and T_3_ O-3-7). Seed yield of greenhouse-grown K-5-8 was also similar to that for Westar.

### Trichome phenotypes

Trichome densities in Arabidopsis vary proximal to distal along the leaf surface and along the leaf edge and show trichome branching. All *B. napus* K-TTG1 and O-TTG1 lines developed in the semi-glabrous Westar background were identical to Westar in trichome density, trichome shape (forming a single unbranched spike), and trichome length (data not shown). Hence these plants were not evaluated further due to greenhouse limitations.

In contrast, seedling leaves and stems of O-TTG1 in the hairy AtGL3^+^*B. napus* (confirmed O plants for 3 generations, including the only fertile line O-3-7) were completely glabrous (Fig. 1c). This glabrous phenotype was unstable, since T_4_ O-3-7-5 and O-3-7-3 plants derived from the two independently generated seed lots showed only hairy leaves in two new trichome phenotypes linked with the new growth phenotypes: one type (only on moderately tall plants) displayed a leaf trichome density which was intermediate between the density of hairy AtGL3^+^ leaves and semi-glabrous Westar leaves (Fig. 1d). The other type (only on very short plants) showed leaf trichome density somwhat higher than Westar, but less dense than on tall T_4_ leaves (Fig. 1d).

**Fig. 2:**
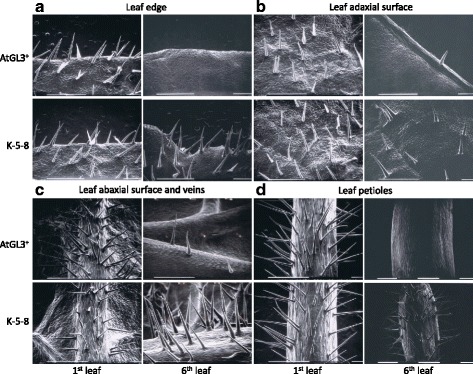
Scanning electron micrographs showing representative differential distribution of trichomes on leaf surfaces of two distinct transgenic hairy canola lines: hairy AtGL3^+^-enhanced *B. napus* and ultra-hairy K-5-8 (*BnTTG1* knock-down in the AtGL3^+^ *B. napus* background). **a**, **c**. First leaf (17 days after germination), and **b**, **d**. Sixth leaf (49 days after germination). White size bars represent 1 mm.

**Fig. 3:**
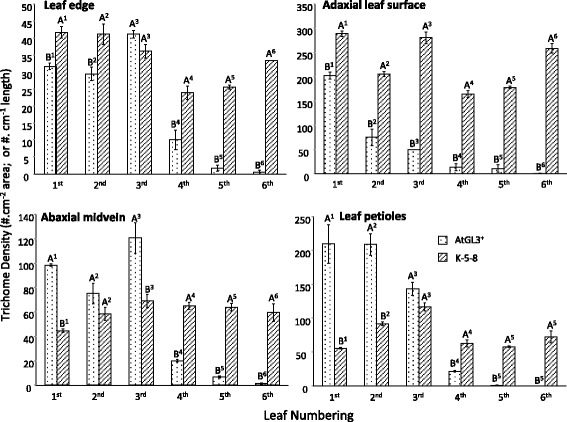
Mean trichome densities across the first to sixth leaves on three week-old seedlings of hairy AtGL3^+^ *B. napus* (AtGL3^+^) and ultra-hairy K-5-8 (*BnTTG1* knock-down in AtGL3^+^ *B. napus*). Leaf edge (cm^−1^); Adaxial leaf surfaces (per cm^−2^); Abaxial surfaces on the mid vein (per cm); Leaf petioles (1 cm length). Trichome densities were measured on three randomly selected positions per leaf surface (for each of three independent plants per line). Seedlings were evaluated two weeks after germination (having first and second leaves) and three weeks after germination (having third to sixth leaves). A Tukey test was carried out between each of the plant lines for each leaf. Significantly different means (± SE) for three plants per line are indicated for each leaf by different numbered letters (*P* ≤ 0.05, comparison is only between individual numbered leaves of the two lines, not between differently numbered leaves)

In K-5-8 and K-6-3, the two TTG1 knockdown plants in the hairy AtGL3^+^*B. napus* background, trichome densities and distribution patterns on young leaves were similar to the hairy AtGL3^+^*B. napus* line, but enhanced after the third leaf stage when trichome density declines in the AtGL3^+^ line (Fig. 2). Trichome densities measured on the edges of the first two leaves of K-5-8 were significantly higher (*P* < 0.05) than in AtGL3^+^*B. napus* plants, while the fourth leaf edges of K-5-8 had more than double the number of trichomes compared to AtGL3^+^*B. napus* (Figs. 2 and 3; not shown for K-6-3). This number increased to over 30 trichomes per cm on the fifth and sixth leaves of K-5-8 (>15-fold increase). K-5-8 plants also produced more trichomes, resulting in higher densities (*P* < 0.05) on the adaxial surface of first to sixth leaves than on AtGL3^+^*B. napus* plants and on the the abaxial leaf mid-vein of the fourth leaf (Figs. 2b, c and 3b, c). In fact, both K-5-8 and K-6-3 produced dense trichome patterning on leaves and petioles up to the 11^th^ or 12^th^ leaf stage, whereas trichome densities decreased on leaves of AtGL3^+^*B. napus* plants after the third leaf. The differences in trichome densities were magnified with leaf expansion, with the leaves of AtGL3^+^*B. napus*, eventually becoming virtually semi-glabrous and similar to leaves of Westar at maturity.

Petioles of AtGL3^+^*B. napus* had higher trichome densities (exceeding 200 per cm petiole length) for first and second leaves compared with K-5-8 (at 60–100), but this declined on the third leaf petiole, with decreasing density up to the sixth leaf stage. Petiole trichome densities for later stage K-5-8 leaves were within the range of those for the first leaf (Figs. 2d and 3d). Furthermore, large numbers of trichomes were found on the stems of K-5-8 and K-6-3 plants above the first node and internode, a phenotype not present in AtGL3^+^*B. napus* plants (Additional file 1: Figure S6). Occasionally trichomes also formed on the peduncle of K-5-8 plants, while this tissue was always glabrous in AtGL3^+^*B. napus* plants (data not shown). This expanded coverage of leaf and stem trichome density was observed through five subsequent generations (T_4_-T_8_) of K-5-8 plants, suggesting a stable phenotype.

**Fig. 4:**
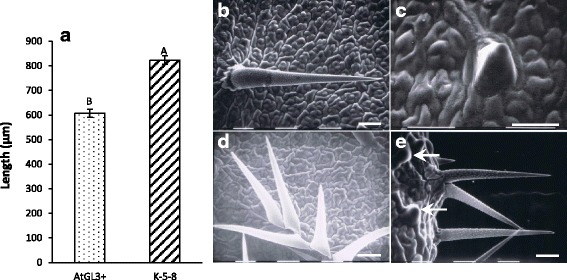
Trichome lengths and morphologies on ultra-hairy K-5-8 and hairy AtGL3^+^ *B. napus*. **a**. Trichome length on first to fourth leaves of three week old seedlings; **b**. Typical fully developed single trichome on K-5-8; **c**. Typical aborted trichome on AtGL3^+^ *B. napus*; **d**. Trichome cluster on K-5-8; **e**. Trichome cluster and typical aborted trichomes (white arrows) on AtGL3^+^ *B. napus*. A Tukey test was carried out between each of the lines. Significantly different means (±SE) for 65 plants per line are indicated by different letters (*P* ≤ 0.05, comparison only between lines). Size bars represent 0.1 mm

K-5-8 and AtGL3^+^*B. napus* produced distinct and different patterns of trichome clustering and trichome morphology. Trichomes were typically unbranched, fully developed, straight, and consistently longer by 100 μm on K-5-8 than those on AtGL3^+^*B. napus* plants (Fig. 4a, b). Short aborted trichomes were produced on leaves of both plant lines, but with a much higher frequency on AtGL3^+^*B. napus* leaves than on K-5-8 leaves, especially after the third leaf (Fig. 4c). Clusters were present approximately two times more frequently on K-5-8 than on AtGL3^+^*B. napus.* The typical number of trichomes in a AtGL3^+^*B. napus* cluster was usually two to three, whereas K-5-8 produced clusters of up to five trichomes (Fig. 4d, e).

### Q-PCR of six trichome regulatory genes using RNA from distinct leaf batches

Q-PCR was conducted on bulked batch RNA samples from 1st to 3rd leaves (batch 1) and 4th to 6th leaves (batch 2) using plant lines with different leaf trichome phenotypes. K-5-8 leaves were hairy in both batches. T_3_ O-3-7 leaves were glabrous in both batches, Westar were semi-glabrous in both batches, and AtGL3^+^*B. napus* plants were hairy only in batch 1, while batch 2 were semi-glabrous. At the time of this experiment, the instability and reversion of the O-3-7 line into two growth and trichome-bearing phenotypes had not surfaced.

**Fig. 5:**
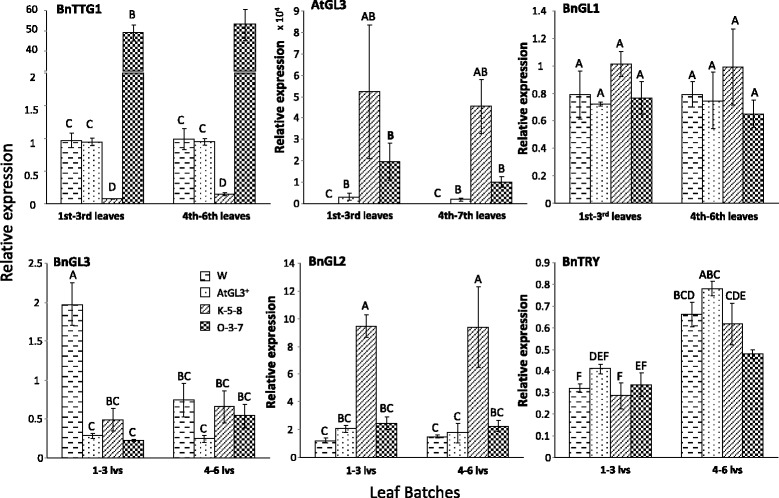
Combined relative expression (representing all orthologues and paralogues) of six trichome regulatory genes by Q-PCR in two leaf tissue batches of seedlings from semi-glabrous *B. napus* cv Westar (W), hairy AtGL3^+^ *B. napus*, ultra-hairy K-5-8 (*BnTTG1* knock-down in AtGL3^+^ *B. napus*), and glabrous T_3_ O-3-7 (*BnTTG1* over-expressed in AtGL3^+^ *B. napus*). Batch 1: 1st to 3rd true leaves; Batch 2: 4th to 6th true leaves. Relative expression levels for *AtGL3*, *BnTTG1*, *BnGL1*, *BnGL2*, *BnGL3*, and *BnTRY* are plotted relative to the expression levels of the same genes in glabrous cotyledons of the *B. napus* cv Westar control line (set at 1; data not shown). The latter (cotyledon) expression was also normalized to the *B. napus* cotyledon expression of the *ACETYL TRANSFERASE 2* (*ACT2*), which was found to be stable across cotyledon and leaf growth stages (relative to the stable *EF1* house-keeping gene). A Tukey test (for each gene) compared the mean relative expression between the four different plant lines and different leaf batches. One cDNA sample per tissue batch per plant was plated into three wells (technical replicates) from each of three plants (biological reps) per line. Cotyledons of five Westar plants (technical reps) were combined to make one control biological replicate. Significantly different means of three individual plants per line (± SE) are indicated by different letters (*P* ≤ 0.05)

Summarized transcript levels were measured for *AtGL3* and the composite of all homologues for each of five *B. napus* trichome regulatory genes, *BnGL1*, *BnGL2*, *BnGL3*, *BnTTG1* and *BnTRY*, known to be involved in MBW tri-protein complex activity in Arabidopsis. Additional file 2: Tables S1B and S1C describe the number of expected gene copies that likely would be measured in *B. napus* batch leaf samples using specific primers designed to detect as many orthologues and paralogues possible for each gene. The relative expression of the *B. napus ACYL TRANSFERASE 2* (*BnACT2*) control gene was stable with low variability (*P* < 0.05) in all leaf batches for all four lines and allowed for comparison across Q-PCR plates (data not shown). As expected, *BnTTG1* over-expression in glabrous O-3-7 leaves was 50-fold higher (*P* ≤ 0.05) compared to Westar and AtGL3^+^*B. napus* leaves and 500-fold higher compared with the K-5-8 line, while the BnTTG1-knockdown line K-5-8 showed >50-fold lower *BnTTG1* transcript levels than both Westar and AtGL3^+^*B. napus* (Fig. 5). The *BnTTG1* primers used in this analysis could potentially have amplified as many as five orthologous copies, but more realistically two as these were more strongly detectable in young 1st leaves by RNA sequencing (see below) (Additional file 2: Table S1B, S2A, S2B), although the batch leaf samples covering more leaf developmental stages may have had a different gene copy number. In contrast, *AtGL3* expression was highly variable and highest in expression in both leaf batches of K-5-8, whereas *AtGL3* expression in glabrous O-3-7 leaves was intermediate between K-5-8 and AtGL3^+^*B. napus* leaves.

Primers for *BnGL3* could only retrieve one *B. napus* sequence from NCBI and none from EST databases or http://brassicadb.org/brad/, but NCBI actually held 5 additional *B. napus* genes that did not show up in the Blast search and two had 100 % primer identity (Additional file 2: Table S1B,C). Moreover, three of these *BnGL3* genes had been detected by RNA sequencing in young leaf tissue. In the batch leaf tissue, relative composite expression of *BnGL3* was constant and relatively low across most lines, although *BnGL3* had somewhat higher expression in Westar. *BnGL1* expression was also constant and low, potentially representing as many as three copies. *BnGL2* expression (regulated by the MBW in Arabidopsis) was low in Westar, AtGL3^+^*B. napus* and O-3-7 leaf batches and 3-fold higher in K-5-8 leaves (Fig. 5). Here, the primers would likely have detected only two *B. napus* copies.

A very low transcript level was detected for *BnTRY* (the negative regulator of trichome initiation) in leaf batch 1–3 (representing young leaves) from all four lines. This level potentially represented the combined expression of as many as four *B. napus TRY* genes (which could be easily detected by young leaf RNA sequencing) (Additional file 2: Table S2A, S2B). However, the only database that retrieved TRY genes was the *B. rapa* database. *BnTRY* batch leaf expression remained consistently low between all four lines (Fig. 5f) regardless of a trichome or glabrous phenotype, but expression increased two-fold in leaf batch 4–6 (representing more mature leaves),

### Trichome-related differential gene expression using RNA sequencing of first leaves

Prompted by the Q-PCR expression patterns of three trichrome regulatory genes (*BnGL3*, *BnGL2*, and *BnTRY*) in several of the batch-leaf Q-PCR reactions, we carried out total RNA sequencing of the first leaf in ultra-hairy K-5-8 and hairy AtGL3^+^*B. napus* relative to semi-glabrous Westar before emergence of the second leaf. This was intended to determine the impact of our gene manipulations on individual *B. napus* homologues of well-known trichome regulatory genes and less-well known trichome genes. At the time of RNA sequencing, the glabrous leaf *BnTTG1* knockdown line (O-3-7) and its revertant plants were not available due to infertility and death of glabrous plants carrying the O-TTG1 construct. A total of 236 trichome genes determined by a recent survey of TAIR and the literature [8] (many commonly known in [2, 61]), were evaluated for changes in expression in AtGL3^+^*B. napus*, K-5-8, and Westar. One hundred (100) trichome genes (37 commonly known) were differentially expressed in K-5-8 relative to Westar, with 61 orthologues (24 commonly known) mapping to *B. oleracea* and 39 orthologues (13 commonly known) to *B. rapa* (using a combined artifical *B. napus* mapping genome) (Additional file 2: Table S2A, S2B). In contrast, only 10 *B. oleracea*-type orthologues (5 commonly known) and five *B. rapa*-type orthologues (one commonly known) showed expression changes in AtGL3^+^*B. napus* leaves.

At this early stage of leaf development (compared with the mixed stages in the batch leaf samples), very few changes in trichome regulatory gene expression were observed in the AtGL3^+^*B. napus* line relative to Westar. Four regulatory genes, including, *B. rapa* and *B. oleracea* orthologues of the *MYB-LIKE 2* (which negatively regulates *GL2* expression in Arabidopsis) [47], one *BoTTG1*, and one *BoGL2* orthologue (with a 2nd *BrGL2* very close to significance; Additional file 2: Table S2B) were strongly (and significantly) up-regulated in the first leaf of AtGL3^+^*B. napus*, while a *BoDUF828* orthologue (with unknown function) and the homeodomain *BrGLABROUS 12* (*BrHDG12*) orthologue were significantly down-regulated (Table 2; remaining trichome genes with significantly different expression relative to Westar found in Additional file 2: Table S2A). Four other *B. napus TTG1* genes were not significantly different (≥0.05) in AtGL3^+^*B. napus* compared with Westar at this leaf stage: Bra009770, Bra029410, Bra029411 (which were barely detectable) and Bra009770 (which was easily detected but numerically much lower in AtGL3^+^*B. napus*) (Additional file 2: Table S2B). Curiously, significant expression differences (*p* ≥ 0.05) were not detected in young AtGL3^+^*B. napus* leaves for two *GL3*s, four *EGL3*s (*MYC2*), three *GL1*s, eight *TRY*s, five *CAPRICE*s (*CPC*s), six *CAPRICE-LIKE*s (*CPL*s, ETC3), and two *ENHANCER OF TRY AND CPC1* (*ETC1*s) relative to Westar leaves, all of which were detectable at least to some degree by RNA sequencing (Additional file 2: Table S2B). However, several genes of these non-significant genes also had higher numerical mean differences in AtGL3+ *B. napus* relative to Westar (Bo8g117860 *ETC1*, Bo9g035460 *EGL1/3*, Bo3g054440 *ETC3*, Bra003535 and Bo4g141980/90 *GL3*s, Bo3g054440 and Bra008539 *CPC*s, and Bo3g022870, Bo9g110930, and Bra022637 *TRY*s), but with very high variability (Additional file 2: Table S2B).

**Table 2:** ^a^Selected differentially expressed trichome-related leaf ESTs detected by RNA sequencing of hairy AtGL3^+^ *B. napus* and ultra-hairy K-5-8 relative to semi-glabrous Westar

Gene ID	Arabidopsis homologue	^b^FPKM Hairy line (Sample 1)	FPKM Westar (Sample 2)	^c^log2 (FC-S1/S2)	P_value
Hairy AtGL3^+^ *B. napus* relative to semi-glabrous Westar					
Bra016164	AT1G71030,ATMYBL2,MYBL2, MYB like 2	91.65	7.27	3.66	5.63E-04
Bo7g096780	AT5G24520,TTG1,TTG,URM23,ATTTG1, Transducin WD40 repeat like superfamily protein	7.26	0.58	3.64	2.95E-03
Bo6g014160	AT1G71030,ATMYBL2,MYBL2, MYB like 2	32.04	2.91	3.46	6.66E-04
Bo6g046840	AT1G79840,GL2, HD ZIP IV family of homeo, leucine zipper protein with lipid binding START domain	7.61	0.86	3.14	9.69E-03
Bo3g025000	AT2G31110, Plant protein of unknown function DUF828	2.20	19.66	−3.16	1.97E-03
Bra016578	AT1G17920,HDG12, homeodomain GLABROUS 12	0.38	6.02	−4.00	2.94E-03
Ultra-hairy K-5-8 relative to semi-glabrous Westar					
Bra003535	AT1G79840,GL2, HD ZIP IV family of homeo, leucine zipper protein with lipid binding START domain	1.60	0.31	2.38	6.03E-03
Bra016164	AT1G71030,ATMYBL2,MYBL2, MYB like 2	88.18	7.27	3.60	0.00E+00
Bo6g014160	AT1G71030,ATMYBL2,MYBL2, MYB like 2	30.33	2.91	3.38	3.95E-10
Bo6g046840	AT1G79840,GL2, HD ZIP IV family of homeo, leucine zipper protein with lipid binding START domain	3.37	0.86	1.96	4.15E-03
Bo2g070770	AT1G71030,ATMYBL2,MYBL2, MYB like 2	10.86	2.86	1.93	3.70E-04
Bo9g083130	AT5G52510,SCL8, SCARECROW like 8	9.11	2.41	1.92	1.45E-09
Bra028312	AT5G52510,SCL8, SCARECROW like 8	5.34	1.61	1.73	9.64E-05
Bo8g080200	AT3G50800, unknown protein	13.53	4.47	1.60	4.35E-03
Bra036862	AT3G50800, unknown protein	24.99	11.63	1.10	2.16E-03
Bra024875	AT2G01570,RGA1,RGA, GRAS family transcription factor family protein	33.57	20.95	0.68	1.11E-03
Bo3g013120	AT5G18410,PIR,KLK,PIR121,SRA1,PIRP,ATSRA1, transcription activators	6.06	9.65	−0.67	1.01E-02
Bo4g039020	AT2G35110,GRL,NAP1,NAPP, transcription activators	9.66	15.68	−0.70	2.44E-03
Bo1g078380	AT3G48750,CDKA;1,CDC2AAT,CDK2,CDC2,CDC2A,CDKA1, cell division control 2	12.99	22.38	−0.78	1.01E-02
Bra018866	AT1G50660, unknown protein	3.55	6.17	−0.80	2.53E-03
Bo6g010070	AT1G73360,HDG11,EDT1,ATHDG11, homeodomain GLABROUS 11	6.82	11.97	−0.81	3.04E-03
Bra009218	AT5G06700,TBR, Plant protein of unknown function DUF828	4.02	7.27	−0.86	7.21E-04
Bo8g077280	AT3G48750,CDKA;1,CDC2AAT,CDK2,CDC2,CDC2A,CDKA1, cell division control 2	8.26	15.62	−0.92	6.49E-04
Bra036731	AT1G33240,AT GTL1,AT GTL2,GTL1, GT 2 like 1 AT1G33240,AT GTL1,AT GTL2,GTL1, GT 2 like 1 AT1G33240,AT GTL1,AT GTL2,GTL1, GT 2 like 1 AT1G33240,AT GTL1,AT GTL2,GTL1, GT 2 like 1	5.78	10.96	−0.92	1.90E-03
Bo2g096060	AT1G80350,ERH3,AAA1,FRA2,LUE1,ATKTN1,KTN1,FRC2, FTR, P loop containing nucleoside triphosphate hydrolases superfamily protein	9.78	19.03	−0.96	8.26E-03
Bra018036	AT3G48750,CDKA;1,CDC2AAT,CDK2,CDC2,CDC2A,CDKA1, cell division control 2	6.23	12.17	−0.97	7.66E-04
Bo8g027460, Bo8g027470	AT1G33240,AT GTL1,AT GTL2,GTL1, GT 2 like 1	11.99	23.41	−0.97	2.44E-03
Bo6g077400	AT1G33240,AT GTL1,AT GTL2,GTL1, GT 2 like 1 AT1G33240,AT GTL1,AT GTL2,GTL1, GT 2 like 1 AT1G33240,AT GTL1,AT GTL2,GTL1, GT 2 like 1 AT1G33240,AT GTL1,AT GTL2,GTL1, GT 2 like 1	20.53	40.86	−0.99	3.56E-05
Bo7g040300	AT3G23590,RFR1,MED33A, REF4 related 1	5.03	10.02	−0.99	2.13E-04
Bra040010	AT1G33240,AT GTL1,AT GTL2,GTL1, GT 2 like 1 AT1G33240,AT GTL1,AT GTL2,GTL1, GT 2 like 1 AT1G33240,AT GTL1,AT GTL2,GTL1, GT 2 like 1 AT1G33240,AT GTL1,AT GTL2,GTL1, GT 2 like 1	9.77	21.52	−1.14	1.30E-05
Bo6g049350	AT3G61150,HDG1,HD GL2 1, homeodomain GLABROUS 1	3.11	7.35	−1.24	4.85E-07
Bra003439	AT3G61150,HDG1,HD GL2 1, homeodomain GLABROUS 1	2.67	6.62	−1.31	8.62E-06
Bo7g116660	AT4G34160,CYCD3;1,CYCD3, CYCLIN D3	1.86	4.68	−1.33	2.01E-03
Bra005177	AT2G37630,ATPHAN,AS1,ATMYB91,MYB91, myb like HTH transcriptional regulator family protein	5.35	13.47	−1.33	1.04E-08
Bo5g021100	AT1G15570,CYCA2;3, CYCLIN A2	6.64	17.09	−1.36	7.19E-06
Bo5g136400	AT3G11540,SPY, Tetratricopeptide repeat TPR like superfamily protein	8.20	21.31	−1.38	2.98E-08
Bra035000	AT3G44200,ATNEK6,NEK6,NIMA never in mitosis, gene A related 6	1.09	2.85	−1.39	1.68E-05
Bra026120	AT1G15570,CYCA2;3, CYCLIN A2	1.96	5.24	−1.42	1.13E-05
Bo8g039430	AT4G14970, unknown protein	0.91	2.52	−1.47	2.17E-03
Bo3g001470	AT5G01360,TBL3, Plant protein of unknown function DUF828	0.69	2.39	−1.79	2.15E-03
Bo7g093130	AT5G28640,AN3,GIF,GIF1,ATGIF1, SSXT family protein	1.55	5.52	−1.83	3.19E-03
Bo1g007590	AT4G34160,CYCD3;1,CYCD3, CYCLIN D3	2.71	10.20	−1.91	1.34E-08
Bra011501	AT4G34160,CYCD3;1,CYCD3, CYCLIN D3	1.27	4.78	−1.92	6.07E-06
Bo8g067530	AT1G17920,HDG12, homeodomain GLABROUS 12	1.26	5.93	−2.24	3.38E-11
Bra022827	AT2G31110, Plant protein of unknown function DUF828	0.73	3.66	−2.32	4.54E-04
Bo3g024610	AT2G30432,TCL1, Homeodomain like superfamily protein	8.99	49.82	−2.47	6.37E-05
Bra016578	AT1G17920,HDG12, homeodomain GLABROUS 12	1.09	6.02	−2.47	1.07E-06
Bra000011	AT2G37630,ATPHAN,AS1,ATMYB91,MYB91, myb like HTH transcriptional regulator family protein	2.77	17.22	−2.64	1.41E-09
Bo3g025000	AT2G31110, Plant protein of unknown function DUF828	1.82	19.66	−3.43	0.00E+00

^a^Genes were selected by screening RNA sequencing data for 50 common trichome genes described in [61]. Expression changes in 65 less common differentially expressed trichome genes are also found in Additional file 2: Table S2A (statistically different expression from Westar) and Additional file 2: Table S2B (not statistically different). ^b^ *FPKM* Fragment Per Kilobase of exon per Million fragments mapped. ^c^log2 FC-S1/S2, log2 of the fold change (hairy leaf sample 1/semi-glabrous leaf sample 2). P_value indicates significant differences of the means of a hairy line relative to the semi-glabrous Westar line

In contrast to AtGL3^+^*B. napus*, 29 trichome-related ESTs were significantly up-regulated and 71 were significantly down-regulated (>2-fold) in the first leaf of K-5-8 relative to Westar (10/31 up/down shown in Table 2. Significantly up-regulated K-5-8 trichome regulatory genes (relative to Westar) included *BoGL2* and *BrGL2* orthologues, three *MYB-LIKE2* orthologues, two *SCARECROW-LIKE 8* orthologues (a protein with unknown function), and the *REPRESSOR OF GIBBERELLIN 1* (*RGA1*, a *VHIID/DELLA* repressor from the *GRAS* family of transcription factors) [48]. Down-regulated K-5-8 regulatory ESTs (relative to Westar) included a *BoPIROGI* gene, a *BoNAP1* transcription factor, copies of the *DUF828* gene, three *CDKs* (*CYCLIN-DEPENDENT KINASE*s), three *CYCD3;1* (*CYCLIN D3*) and two *CYCA2;3* genes, *HOMEOBOX-LEUCINE ZIPPER PROTEIN* genes (two *HDG11*s and one *HDG1*), and two *MYB91* (*MYB DOMAIN PROTEIN* 91 from the *HTH* transcription family). Significant expression differences (*p* ≤ 0.05) were not detected in K-5-8 first leaves (relative to Westar) for 35 common regulatory genes. These non-significant expression patterns included the five *TTG1* genes (unexpectedly), as well as two *GL3*s, four *EGL3*s (*MYC2*), three *GL1*s, eight *TRY*s, five *CAPRICE*s (*CPC*s), six *CAPRICE-LIKE*s (*CPL*s), and two *ENHANCER OF TRY AND CPC1* (*ETC*s), all of which were detectable but some with very low expression. Again, several of these non-significant genes also had numerically different means in K-5-8 relative to Westar, (Bo7g096780 *TTG1*, Bo8g117860 *ETC1*, Bo9g035460 and Bra027796 *EGL3*s, three *CPC*s, three *CPL*s, Bra025508 and Bo4g141980/90 *GL3s*, and Bo9g110930 *TRY* were higher and Bo2g046050 and Bra022637 *TRY*, Bo7g090950 *GL1*, and Bo9g029230 and Bra027653 *EGL*s lower in K-5-8), but with very high variability (Additional file 2: Table S2B).

### Global transcriptome analysis of metabolic, structural, regulatory, and redox genes

**Fig. 6:**
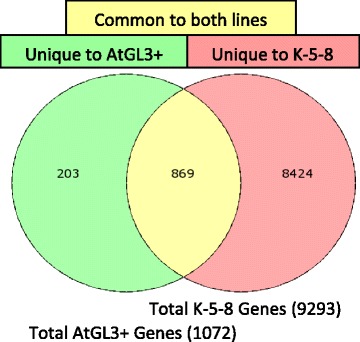
Venn diagram representing the total number of differentially expressed genes (DEGs) in first leaves of hairy AtGL3^+^ *B. napus* and ultra-hairy K-5-8 relative to semi-glabrous *B. napus* cv. Westar leaves. Number within the over-lapping region represents the number of DEGs with differential expression common to the two trichome-enhanced leaf samples. The non-overlapping regions show the number of DEGs unique to each sample

Global changes in metabolite gene expression patterns obtained from RNA sequencing were also compared across the first leaves of AtGL3^+^*B. napus*, K-5-8 and Westar. A massively higher number of genes (nine-fold greater) were significantly differentially expressed in K-5-8 (9293) compared with AtGL3^+^*B. napus* (1072) (each relative to Westar control leaves) (Fig. 6). Of the 1072 differentially expressed EST set for AtGL3^+^*B. napus*, 869 (81 %) were also common to K-5-8, while 203 were unique to the AtGL3^+^*B. napus* transcriptome. In contrast, 8,424 ESTs were differentially expressed only in K-5-8, with the proportion of up and down-regulated genes being equal.

**Fig. 7:**
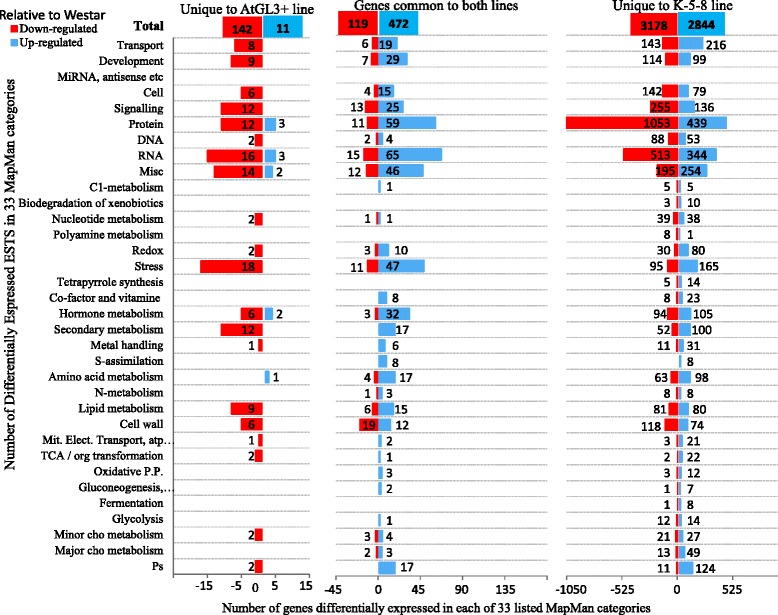
Histogram illustrating numbers of differentially expressed genes (with >2-fold difference) determined by RNA sequencing of first leaves of transgenic hairy (AtGL3^+^) and ultra-hairy (K-5-8) *B. napus* lines (relative to semi-glabrous *B. napus* Westar leaves) in 33 MapMan functional categories (Bins). Data was collected by RNA sequencing. Only genes with >2-fold significantly different transcript levels relative to *B. napus* cv. Westar at *p* < 0.05 are indicated. Red indicates numbers of down-regulated genes. Blue indicates numbers of up-regulated genes

A broad spectrum of differentially expressed metabolic genes not previously noted as targets of the *GL3* or *TTG1* transgenes was identified by mapping the EST sets to 34 MapMan functional categories (Fig. 7). Total down-regulated and up-regulated genes and major functional categories clearly showed that the vast majority of transcript changes occured in K-5-8 leaves. These major categories included protein signalling, RNA, cell transport and development, whereas transcript levels for very few genes in these categories were altered in AtGL3^+^*B. napus* (Fig. 7; Additional file 1: Figure S7A, S7B). Instead, down-regulation of five ethylene pathway genes, three ERF homologues (ethylene responsive elements), and three genes in the auxin pathway was observed in AtGL3^+^*B. napus* (Additional file 2: Table S10). In addition, transcription factors known to regulate plant growth were down-regulated in AtGL3^+^*B. napus* relative to *B. napus* Westar , while a host of such TFs were up-regulated and down-regulated in K-5-8 (Additional file 1: Figure S7A,S7B; Additional file 2: Table S18). Up-regulated K-5-8 TFs included those coding for multiple zinc finger proteins (eg. AN1-like and a salt inducible protein), basic helix loop helix (*bHLH*) DNA binding proteins, developmental proteins (eg. AGAMOUS like 46), MYB proteins, flowering proteins (eg. CONSTANS like), and WRKY family transcription factors. Down-regulated K-5-8 TFs included MYB-like factors (eg. *MYB111*, *MYB91*, *HTH*) and bHLHs, among others.

Expression of cell wall carbohydrate and protein genes was also altered in both AtGL3^+^*B. napus* and K-5-8; but again, far more of these genes were differentially expressed in K-5-8, with only six uniquely upregulated in AtGL3^+^*B. napus* (Additional file 2: Table S10). K-5-8 also had more dramatic changes in expression levels of ESTs involved in growth and development, photosynthetic light reactions, Calvin cycle, amino acid synthesis, mitochondrial electron transport, cell wall and lipid synthesis, with *ATKCR1* (beta ketoacyl reductase), *KCS* (ketoacyl CoA synthase) and *CER1* (fatty acid hydroxylase superfamily) involved in wax regulation only affected in K-5-8 (Fig. 7; Additional file 1: Figure S7A, 7SB; Additional file 2: Table S12).

In genes specifying redox pathways, two plant ascorbate oxidase genes were differentially expressed only in AtGL3^+^*B. napus*, while the remainder of redox ESTs predominantly were found in K-5-8 leaves (Additional file 2: Table S15). Genes specifying secondary metabolism were also differentially expressed in young *B. napus* leaves. In particular, 184 K-5-8 ESTs were mapped to i) flavonoids and phenylpropanoids categories (Additional file 2: Table S4) ii) lignans and cell wall lignin (Additional file 2: Tables S4 and S5), iii) terpenoids (Additional file 2: Table S7), iv) simple phenolics (Additional file 2: Table S5), v) shikimates (Additional file 2: Table S6), vi) S-metabolism (glucosinolates and nitriles) (Additional file 2: Table S8), and vii) N-metabolism (alkaloids) (Additional file 2: Table S9) (Additional file 1: Figure S7A). In contrast, only 12 differentially expressed secondary metabolite ESTs were identified in AtGL3^+^*B. napus* leaves relative to Westar and the 10 flavonoid ESTs increased in AtGL3^+^*B. napus* were unchanged in K-5-8 ((Additional file 1: Figure S7B; Additional file 2: Table S3). Moreover, *PAL4* was up-regulated in K-5-8 and transcripts for two copies of *PAL2* were 2-fold less compared with Westar leaves (Additional file 2: Table S4). In contrast, expression of *PAL1* and *PAL2* was upregulated in AtGL3^+^*B. napus* compared to Westar. Transcript levels for *CHS*, *DFR*, and *ANS* were confirmed via Q-PCR [42].

### Q-PCR of individual redox, cell wall, and trichome gene orthologues in first true leaves

Q-PCR analysis was conducted on five redox, four cell wall carbohydrate, two lignin, and eight trichome regulatory genes and one trichome cell wall gene using first true leaves of semi-glabrous Westar, hairy AtGL3^+^*B. napus*, ultra-hairy K-5-8, and T_4_ O-3-7 plants. These O-3-7 plants were derived from seeds of fully glabrous T_3_ plants (Fig. 1c), but the T_4_ O-3-7 plants showed partial “reversion” into two types of growth phenotypes, each linked with trichome-bearing leaves. Q-PCR was conducted only 1st leaves of larger T_4_ O-3-7 “revertant” plants with intermediate trichome density, since the very small T_4_ O-3-7 growth phenotype with lower trichome density was unhealthy and died before sufficient leaf tissue could be harvested for RNA extraction (Fig. 1d) and T_3_ glabrous plants were no longer available to test (having been used for batch leaf Q-PCR and seed development). The purpose of this Q-PCR experiment was to determine whether trichome and growth gene expression profiles and phenotypes in first leaves were consistent with Q-PCR expression patterns for trichome genes in leaf batches (Fig. 5) and RNA sequencing in first leaves (Table 1; Additional file 2: Tables 2SA; 2SB), particularly for *BnTTG1* knockdown in K-5-8 and over-expression in O-3-7.

**Fig. 8:**
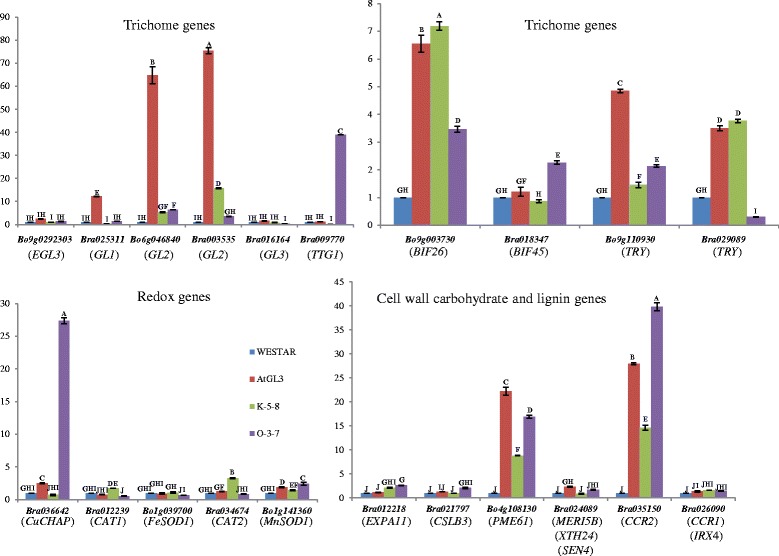
Relative expression (by Q-PCR) of individual orthologous genes for a range of redox, cell wall carbohydrate, lignin, and trichome genes in young seedling first leaves of semi-glabrous *B. napus* cv Westar (W), hairy AtGL3^+^ *B. napus*, ultra-hairy K-5-8, and moderate semi-glabrous revertant leaves of T_4_ O-3-7-5. O-3-7-5 was grown from seed harvested from glabrous leaf O-3-7. *CuCHAP*, Cu chaperone for Superoxide Dismutase 1 (*SOD1*); *CAT1,* Catalase 1; *FeSOD1,* Fe Superoxide Dismutase 1; *CAT2*, Catalase 2; *MnSOD1*, Mn Superoxide Dismutase 1; *EXPA11,* Expansin 11; *CSLB3*, Cellulose synthase-like B3; *PME61*, Pectin methylesterase 61; MERI5*B*, *XTH24*; *SEN4*, Xyloglucan endotransglucosylase hydrolase; *CCR2*, Cinnamoyl CoA reductase 2; *CCR1*, Cinnamoyl CoA reductase 1; *EGL3*, Enhancer of Glabra 3; *GL1*, Glabra 1; *GL2*, Glabra 2; *TTG1*, Transparent testa glabra 1; *BIF26*, Bi-refringence 26; *BIF45*, Bi-refringence 45; *TRY*, Triptychon

Q-PCR expression analysis of the *BnTTG1 Bra*009770 orthologue was extremely high in the moderately hairy O-3-7 revertant leaves and very low in ultra-hairy K-5-8 leaves (Fig. 8). Expression of *Bra*009770 in Westar and the AtGL3^+^*B. napus* line was numerically higher than in K-5-8 at this leaf stage but not significantly different. This Q-PCR result was consistent with the lack of significantly different expression for this gene in Westar, AtGL3^+^*B. napus*, and K-5-8 first leaves by RNA sequencing (Additional file 2: Table S2B). It also confirmed the continued presence of a viable *BnTTG1* over-expression construct in the remaining O plants regardless of the “reversion” to a trichome phenotype. *BnEGL3* Bo9g029230 and *BnGL3* Bra016164 orthologues also showed very low Q-PCR expression, with no significant differences of the means between the four lines (consistent with RNA sequencing), although expression of these two trichome genes was numerically lower in K-5-8 in the latter experiment.

In contrast, Q-PCR-based expression levels for *BnGL1* Bra025311, the two *BnGL2* orthologues Bo6g046840 and Bra003535, and the two *BnTRY* orthologues Bo9g110930 and Bra029089 were 10-, 63-, 73-, 5- and 3-fold higher, respectively, in hairy AtGL3^+^*B. napus* first leaf relative to semi-glabrous Westar (Fig. 8). This was consistent with RNA sequencing in which *BnGL2* Bo6g046840 was significantly higher, *BnGL2* Bra003535 almost significantly higher, and *BnGL1* Bra025311 and *BnTRY* Bo9g110930 numerically higher (but with high variability) in AtGL3^+^*B. napus* (Additional file 2: Table S2A; S2B). In the ultra-hairy K-5-8 and moderately hairy O-3-7-5 leaves, Q-PCR expression of the *BnGL1* Bra025311 was identical to Westar (consistent with RNA sequencing patterns). However, the two tested *BnGL2* orthologues showed significantly different Q-PCR expression patterns when both genes were compared with each other in the two constructed lines, such that *BrGL2* Bo6g046840 was higher in ultra-hairy K-5-8 compared with moderately hairy O-3-7-5 first leaves, but the *BoGL2* Bra003535 orthologue was identical in expression level between these two lines. Both *GL2* genes were intermediate expressors in these two constructed lines compared with semi-glabrous Westar and hairy AtGL3^+^*B. napus* (Fig. 8). Moreover, the two *BnTRY* test orthologues Bo9g110930 and Bra029089 showed inverse expression patterns in ultra-hairy K-5-8 compared with moderately hairy O-3-7-5 first leaves (Fig. 8). One trichome-specific structural gene, *BIREFRINGENCE 26* (*BIF26*, Bo9g003730), proposed to specify cell wall polysaccharide O-acetyltransferase, showed a higher expression pattern in AtGL3^+^*B. napus*, K-5-8, and O-3-7-5 relative to Westar consistent with trichome phenotypes (and RNA sequencing of three of these lines), but this correlation did not hold true for *BIF45* (Bra018347) (Fig. 8).

Four redox genes, *CATALASE1* (*CAT1*, Bra012239) *CAT2* (Bra034674), *Fe SUPEROXIDE DISMUTASE1* (*FeSOD1*, Bo1g039700), and *MnSOD1* (Bo1g141360), three cell wall carbohydrate genes, *EXPANSIN11* (*EXPA11*, Bo7g061700), *CELLULOSE SYNTHASE-LIKE B3* (*CSLB3*, Bra021797), and *XYLOGLUCAN ENDOTRANSGLUCOSYLASE HYDROLASE* (*MERI5B*, *XTH24*, or *SEN4*, Bra024089), and the lignin gene *CINNAMOYL CoA REDUCTASE1* (*CCR1, IRX4*, Bra026090) were expressed at low levels (by Q-PCR) in first leaves of all four tested lines (Fig. 8). Of these low-expressing genes, the *BrCAT1* orthologue showed significantly higher expression in first leaves of the most robust line (K-5-8) and lower in less robust O-3-7-5 relative to robust Westar, while expression in the less robust AtGL3^+^*B. napus* line was closest to the O-3-7-5 line. *BrCAT2* and *BoFeSOD1* orthologues also showed highest overall expression in K-5-8 leaves and lowest to O-3-7-5 (although differences with the other two lines were not always significant). Moreover, Q-PCR expression levels for the low-expressing orthologue *BraXTH24* and three high-expressing orthologues for *COPPER CHAPERONE for SOD1* (*CuCHAP*, Bra036642), *PECTIN METHYLESTERASE* (*PME61*, Bo4g108130), and *CCR2* (Bra035150) were consistently higher in the two less robust lines compared with robust lines (Fig. 8). In contrast, a reverse Q-PCR expression pattern occurred for low-expressing *BoMnSOD1* and *BraCSLB3*, such that their expression was highest in O-3-7-5 leaves and lowest in K-5-8 leaves, while expression levels for *CCR1* were no different for the four lines and *EXPA11* expression was inconsistent with growth phenotypes.

The *CAT1*, *CAT2*, *EXPA11*,_and *PME61* Q-PCR patterns for first leaves were consistent with RNA sequencing expression patterns for AtGL3^+^*B. napus*, and K-5-8 relative to Westar, while *FeSOD1*, *MnSOD1*, *CuCHAP*, *CSLB3*, *XTH24*, *CCR2* patterns were inconsistent between the two expression techniques (Additional file 2: Tables S4 S10, S15). Overall relative expression levels of *FeSOD1*, *CAT2*, *PME61*, and *CCR2* compared with *CAT1*, *MnSOD*, *CSLB3*, and *CCR1* were also inconsistent between the two experiments.

## Discussion

### Mis-expression of *BnTTG1* together with *AtGL3* affects growth and trichome patterning

*TTG1* is a WD40 trichome regulatory gene known to have pleiotropic effects in *Arabidopsis* [49]. Complexed with GL3 and MYB proteins, TTG1 directly regulates trichome patterning on *Arabidopsis* leaves [50]. However, very little is known on how *GL3* and *TTGI* impact plant development and trichome patterning in Brassicas.

In this study, expression level of *BnTTG1* was manipulated in two ways in a AtGL3^+^*B. napus* ‘Hairy Canola’ background [35]). This resulted in pleiotropic effects on plant growth and development (especially trichome development). Earlier generations of the AtGL3^+^*B. napus* background line had shown some depression of growth and fertility, which had lessened in severity such that this background line was stable and only slightly shorter in stature and a week later in development compared to *B. napus* Westar. Knockdown of *BnTTG1* in this hairy AtGL3^+^*B. napus* background restored normal (Westar-like) greenhouse growth and development to stable ultra-hairy K-6-3 and K-5-8 lines. In contrast, over-expression of *BnTTG1* in this same hairy background resulted in a shorter, very low-fertility glabrous T_3_ homozygous phenotype (O-3-7). This only remaining O-line was unstable, since it generated two new T_4_ phenotypes (moderately large plant with moderate trichome density and very small plant with trichome density somewhat higher than semi-glabrous Westar). Altered growth or trichome phenotypes were not observed in *B. napus* Westar background plants over-expressing *BnTTG1* or knocked down in this gene, but these latter plants are still heterozygous and could be masking phenotypes we were unable to detect.

The restoration of a normal Westar growth phenotype in ultra-hairy K-5-8 and K-6-3 down-regulated in *BnTTG1* expression and the opposite growth and trichome phenotype in O-3-7 suggests that a combination of high levels of *GL3* and *TTG1* transcripts negatively impacts *B. napus* growth above and beyond when *GL3* transcription alone is enhanced. This is supported by reduced transcript levels for select cell wall, hormone, and wax genes in AtGL3^+^*B. napus* leaves (relative to Westar leaves), but much more extreme changes in gene expression levels and gene diversity in K-5-8 leaves for genes involved in growth and development, photosynthetic light reactions, Calvin cycle, amino acid synthesis, mitochondrial electron transport, cell wall, and lipids (relative to Westar). These expression and phenotype data infer a window of *TTG1* expression levels that maintains optimal growth and is supported by the fact that K8 and K10 plants within the same Bn-TTG1 knockdown transgenic series, but having less extreme reductions in *BnTTG1* transcript levels, maintain an unaltered AtGL3^+^*B. napus* growth and trichome phenotype.

Expression of *AtGL3* in *B. napus* and RNAi knockdown or overexpression of *BnTTG1* within the AtGL3^+^*B. napus* background used transgene constructs under the control of a strong constitutive promoter (*35S*_*p*_). This would lead to mis-expression of these two trichome regulatory genes outside of their usual expression range, developmental time, and tissue, and would simultaneously affect other trichome genes/proteins they normally do not regulate when controlled by their own promoters. As well, it would affect other genes and proteins that impact on many different cellular factors, including those which affect growth and plant health. *In silico* microarray data from the eFP browser (http://bar.utoronto.ca/efp/cgi-bin/efpWeb.cgi*.)* and the TileViz browser (http://jsp.weigelworld.org/tileviz/tileviz.jsp*)* shows that the closely related *AtTTG1* is normally expressed throughout Arabidopsis (a closely related species) over a range of developmental stages and tissues (Additional file 1: Figure S9A) and usually has 10-fold higher expression than *AtGL3* (Additional file 1: Figure S9B), which normally has a more limited expression [51–53]. This type of complete developmental expression map for the main trichome regulatory genes and different biochemical, growth, and health genes would be very useful to develop in a crop species like *B. napus*, since it would give us additional tools with which to analyze the impact of manipulating these (and other) genes for practical purposes. Mis-expression is a main feature of all transgenes under *35S*_*p*_ control, but the impact of mis-expression would be much broader using a regulatory transgene than a structural gene, which would have a more limited biochemical impact (eg. on only one or two pathways). However, there is still a limited possibility that some type of interaction has occurred between the two *35S* promoters themselves to cause the phenotypes observed in K-5-8, K-6-3, and O-3-7. The fact that we recovered two independent transgenic events with K-5-8 phenotypes suggests that at least the *BnTTG1* knockdown within the AtGL3^+^ background can generate the same stable phenotypes regardless of having two 35S promoters within the same germplasm.

The low viability and poor seed quality and yield of the O-type *BnTTG1* over-expression within the AtGL3^+^ background and the instability of the growth and trichome phenotypes in the T_4_ generation shows that an over-abundance of *BnTTG1* and *AtGL3* transcripts negatively impacts on plant health and stability. In a range of Arabidopsis backgrounds, seedling lethality has been shown to result from the combined over-expression of *GL1* together with a maize bHLH *R* gene, although *GL1* enhancement by itself has no effect on viability [54]. The most vigorous of these latter Arabidopsis plants are homozygous *ttg1*. Hence, it appears that growth and development in both *B. napus* and Arabidopsis are sensitive to the proportion and level of *TTG1* and *bHLH* (eg. *GL3*) expression.

### *BnTTG1* and *BnTRY* may have different roles in *B. napus*

*AtTTG1* over-expression in Arabidopsis does not increase trichome density above the background level [13] even though Arabidopsis *ttg1* null mutants initiate very few trichomes [15]. Similarly, our *B. napus* studies showed that over-expression or knockdown of *TTG1* in the semi-glabrous Westar background had little to no effect on trichome density, although these plants are still heterozygous and should eventually be re-evaluated in homozygous lines. In contrast, over-expression of *BnT1* in the hairy AtGL3^+^*B. napus* background (i.e., producing the O-3-7 line) produced fully glabrous leaf and stem phenotypes, although the glabrous leaf phenotype was unstable in the only surviving T_3_ seed line when T_4_ plants showed a “reversion” to trichome densites intermediate between the semi-glabrous Westar leaf and the hairy AtGL3^+^ leaf. However, knockdown of *TTG1* in this hairy AtGL3^+^*B. napus* background produced stable plant lines (K-5-8 and K-6-3) with broader trichome coverage, higher trichome densities, and longer trichomes, i.e., ultra-‘Hairy Canola’. These opposite trichome phenotypes produced when using the hairy AtGL3^+^ background strongly suggest that the high transcript level for *TTG1* in leaves of Westar negatively impacts trichome development (and potentially may also in other *B. napus* cultivars, depending on the level of *GL3* transcription). In Arabidopsis, transcription of the trichome outgrowth gene *GL2* is controlled by a tri-protein activator complex GL1-GL3-TTG1, with GL1 and GL3 binding directly to the *GL2* promoter [19]. While AtTTG1 does not bind directly to the *GL2* promoter, it is thought to be a positive regulator necessary for stabilizing Arabidopsis GL1-GL3 di-protein complexes through its association with GL3. Instead, our Q-PCR results support the role of *BnTTG1* as a negative regulator of trichome initiation in *B. napus* by the decreased expression of *GL2* in Q-PCR batch leaf experiments when an elevated *GL3* level (in AtGL3^+^*B. napus*) was coupled with over-expression of *BnTTG1* (as in glabrous O-3-7).

Manipulation of *BnTTG1* transcript levels appeared to raise the Q-PCR batch leaf transcript level of *AtGL3* in both the glabrous O-3-7 over-expression plants and the ultra-hairy K-5-8 plants compared with Westar and the AtGL3^+^*B. napus* plants, even though *BnTTG1* batch leaf transcript levels were completely opposite in these K-5-8 and T_3_ O-3-7 plants. In fact, the levels of *AtGL3* were much higher in K-5-8 leaf batches than in AtGL3^+^*B. napus* leaf batches. Moreover, transcript levels for composite and individual *BnGL2* orthologues were much higher for AtGL3^+^*B. napus* than for K-5-8 in both leaf batches and first leaf Q-PCR experiments even though trichome length and coverage were only expanded in K-5-8. These expression patterns should be explored in more detail using two ultra-hairy lines (K-5-8 and K-6-3) and by replacing the *AtGL3* gene with a range of Brassica *GL3* orthologous genes to determine whether these expression patterns are plant-specific, gene-source specific, or a more generalized pattern.

Trichome initiation is not affected in Arabidopsis *gl2* mutants, although shorter aborted trichome cells expand laterally over the leaf surface [19]. Similar short trichomes are also observed in an Arabidopsis *gl3* mutant [55]. Thus, the Arabidopsis model suggests that increased expression of *GL2* (and *GL3*) is required for proper outgrowth of trichomes [19]. While *tt1g* mutations in reduce *GL2* expression, expression of a *35S::R* (*bHLH*) transgene in a *ttg1* Arabidopsis background increases *AtGL2* expression [56]. Similarly, an elevated *AtGL3* expression in Arabidopsis bypasses the TTG1 requirement to induce *GL2* expression [56]. Likewise in *B. napus*, higher expression of *GL2* occurs in leaf batches (but not first leaves) of K-5-8 relative to AtGL3^+^*B. napus*, and this batch leaf expression profile correlated with increased trichome length. However, aborted trichomes on the leaf surface of AtGL3^+^*B. napus* did not expand laterally along the leaf surface, as in Arabidopsis. Instead, their growth was arrested immediately in *B. napus* after trichome initiation. These observations, together with the invariable (composite) *GL1* expression found in batch leaf Q-PCR analysis of all four *B. napus* line, suggest that *BnGL2* also controls *B. napus* trichome length and out-growth in *B. napus* and that strong *TTG1* transcription together with strong *GL3* expression inhibits this process.

Clusters of trichomes are found along the leaf margins of weak alleles of Arabidopsis *try* and *ttg1* mutants [57]. K-5-8 also showed trichome clustering on leaf margins, suggesting a role for *BnTTG1* in the lateral inhibition of trichome formation in neighbouring cells of *B. napus*. The composite weak expression of *BnTRY* in both glabrous and trichome-bearing Q-PCR leaf batches, as well as the stronger expression of *BnTRY* orthologues in first leaves ultra-hairy K-5-8 and hairy AtGL3^+^ plants suggest that *TRY* may have functions other than inhibiting trichome initiation in the Brassicas. This hypothesis is supported by the high expression of *BvTRY* in leaves with a dense coverage of trichomes in ultra-hairy *B. villosa* (relative to Westar) [33, 34].

### High expression of the *AtGL3* affects transcription of *BnGL3*

Arabidopsis *AtGL3* expression is controlled by an autoregulatory feedback loop, such that it binds to its own promoter to negatively regulate its own gene expression [58]. In our *B. napus* experiments, over-expression of the *AtGL3* transgene resulted in decreased (composite) expression of *BnGL3* compared to Westar, suggesting that the *B. napus GL3* promoter may be negatively regulated by an excess of *AtGL3*. Amino acid alignments between *AtGL3* and its five *BnGL3* homologues show a range of homologies (69–77 %) that may impact on whether or not the BnGL3 proteins can also bind to the *BnGL3* promoter. (Additional file 1: Figure S8; Additional file 2: Table S1D). The GL3 proteins might also compete for binding sites on G*L2* promoters [56]. Screening *BnGL3* proteins for sequences with binding capability to *BnGL3* and *BnGL2* promoters by conducting one-hybrid and bimolecular binding studies are next steps that could lead towards an understanding of how similar or distinct the *B. napus* trichome regulatory circuit is compared with the Arabidopsis model.

### *BnTTG1* knockdown in an *AtGL3*-enhanced background affects a host of metabolite pathways in *B. napus*

Arabidopsis bHLH genes, such as *GL3*, *EGL3*, *AtMYC1*, and the flavonoid gene *TRANSPARENT TESTA 8* (*TT8*), are intertwined with *TTG1* in several pathways. They participate in tri-protein complexes [59], and they also regulate late-stage flavonoid biosynthesis genes [60–62]. In Arabidopsis, over-expression of *AtGL3* in a wild type background up-regulates expression of the structural genes *DIHYDROFLAVONOL 4-REDUCTASE* (*DFR*) and *ANTHOCYANIDIN SYNTHASE* (*ANS*) from the flavonoid pathway. The promoters of both *DFR* and *ANS* are activated in Arabidopsis by the MBW complex, and expression of both genes is down-regulated in *ttg1* mutants [61, 62]. Similarly, strong expression of *35S*_*p*_*::AtGL3* in *B. napus* resulted in increased red pigmentation in seedlings (data not shown) and transcription of genes in the flavonoid pathway, but interfered somewhat with plant growth. Growth was even more negatively impacted by strong *AtGL3* expression together with an up-regulation of *BnTTG1* in O-3-7.

Coupling strong *AtGL3* enhancement with the knockdown of *BnTTG1* (K-5-8) resulted in the decline of leaf flavonoid transcript levels to Westar levels or lower and fully restored growth. More important, the combined impact of *AtGL3* expression and decreased *BnTTG1* transcription in K-5-8 had a massive impact on the number (and expression intensities) of differentially expressed genes from a broad range of metabolic pathways producing phenylpropanoids, simple phenolics, shikimates, isoprenoids, terpenes, glucosinolates (and breakdown products),alkaloids, structural carbohydrates, lignin, pectin, and redox proteins important for cell wall growth, plant health, and stress protection. Opposite Q-PCR expression patterns were found for several of these genes between 1st true leaves of K-5-8 and T_4_ O-3-7-5 “revertants” or between other growth phenotypes. In contrast, the addition of the *AtGL3* gene alone had far less impact on leaf gene expression, such that only 142 unique genes and 119 DEGs were down-regulated and 11 unique DEGs and 472 common DEGs up-regulated in AtGL3^+^*B. napus* leaves compared with Westar control leaves, while the K-5-8 leaves had a reduction of 3,178 unique DEGs and an induction of 2,844 unique DEGs. Potentially, BnTTG1 protein titre is reduced in K-5-8 leaves, a situation which may positively affect (i.e., increase) the availability of a large portion of GL3 protein tied up (in some manner) in AtGL3^+^*B. napus* and able to impact these other biochemical pathways. Studies on AtGL3 and BnTTG1 protein availability and titre in non-trichome bearing cells of these unique lines may prove fruitful in understanding the mechanism by which these two genes interact to modify growth and metabolism.

## Conclusion

This study is the first to identify the involvement of *BnTTG1* knockdown (with *AtGL3* mis-expression) in restoring growth and enhancing trichome density, coverage, and length and impacting the transcription of many different biochemical and transcription pathways in *B. napus*. It also highlights transcription factors and other gene targets which could be tested in bimolecular complementation experiments and DNA binding studies to identify components that underly this broad effect on cell metabolism and growth. The fact that over-expression of *BnTTG1* in the AtGL3^+^ background is highly lethal and represses trichome development (even though the glabrous phenotype is eventually unstable), supports our hypothesis that *TTG1* inhibits trichome development in *B. napus*. The seed lines generated from this study represent valuable germplasm with which to determine the nature of the instability of these latter plants. Since the growth of the new ultra-hairy *B. napus* canola is now restored and trichome coverage is enhanced compared with the original AtGL3^+^ hairy canola, the two novel plant lines (K-5-8 and K-6-3) should now be advanced into field trials to test their agronomic characteristics and insect tolerance.

## Methods

### Genetic Background Material

*Brassica napus* (cv. Westar) seeds were obtained from the Brassica collection at the Saskatoon Research Center, Agriculture and Agri-Food Canada (AAFC-SRC). The homozygous “Hairy Canola” AtGL3^+^*B. napus* line expressing a *35S*_*p*_*::AtGL3* construct [35] was produced previously in the Gruber lab (AAFC-SRC).

### O-TTGI (over-expression) and K-TTGI (knock-down) cassette construction

*Over-expression*: *BnTTG1* sequences for *B. napus* isoform 1 (EF1175930, called *BnTTG-1*) and *B. napus* isoform 2 (EF175932, called *BnTTG1-2*) were aligned to *B. rapa BrTTG1* (HM208590) from Genbank (Additional file 1: Figure S1A). Based on this alignment, a full-length *BnTTG1-1 CDS* was amplified from total RNA/first-strand cDNA using a commercial RNA-Easy^®^ mini-kit (Qiagen, Valencia, CA, USA) and SuperScript^TM^ II reverse transcriptase (Invitrogen, Carlsbad, CA, USA) and young *B. napus* cv. Westar leaves with specific primers (OFTTG1 and ORTTG1) designed using software at http://www.ncbi.nlm.nih.gov/tools/primer-blast/ and synthesized at Integrated DNA Technologies, Inc. (Coralville, IA, USA) (primer sequences detailed in Additional file 1: Figure S1A and Additional file 2: Table S1A). The PCR product was purified using a QIAquick^®^ PCR purification kit (QIAGEN, Hilden, Germany), cloned into the pGEM^®^-T Easy Vector System (Promega, Wisconsin, USA) in *E. coli* (DH10-β), and sequenced at the National Research Council (Saskatoon). Plasmids were extracted using a QIAprep^®^ Spin Miniprep Kit (QIAGEN) and digested with restriction enzymes. The transgene over-expression cassette was then cloned into the BamHI/SacI sites between the 35S promoter (*35S*_*p*_) and the *NOS* terminator of the binary vector p73-109 to produce O-TTG1 (Additional file 1: Figure S1B).

*Knock-down*: To silence *BnTTG1*, a unique 260 bp region (Additional file 1: Figure S1C) conserved only between *BnTTG1-1* and *BnTTG1-2* was amplified using specific primers LFTTG1/LRTTG1 and RFTTG1/RRTTG1 (detailed in Additional file 2: Table S1A). To confirm the unique nature of this 260 bp sequence, a more recent BLAST to the NCBI database also retrieved only these two *BnTTG1* genes, as represented by six ESTs; EF175930, EF175929.1, EF192030.1, EF175932.1, EF175931.1, and EF192031.1. The fragment was then cloned in sense and antisense orientations into the GUS-intron-containing intermediate vector pBI121 (Clontech, California, USA) at unique XbaI/BamHI and SnabI/SacI sites. The resulting sense-intron-antisense RNAi cassette was subcloned into the unique XbaI/SacI sites between the *35S*_*p*_ and the *NOS* terminator of the binary vector p73-109 to produce K-TTG1 (Additional file 1: Figure S1B).

### Plant transformation

*Agrobacterium tumefaciens* (GV3101) was transformed with O-TTG1, K-TTG1 or the p73-109 empty vector control cassette. Cassette-positive colonies were used to transform *B. napus* cv Westar or “Hairy Canola” (AtGL3^+^*B. napus*) plants using the hypocotyl co-cultivation method developed by [41]. Green calli on 5 mgL^−1^ L-phosphinothricin (PPT) were transferred onto solid shoot regeneration medium every 2 weeks and grown in a controlled growth room at 22/20 °C in a 16/8 h light/dark photoperiod (80 μE.m^−2^.s^−1^) until young leaves developed, then calli with shoots were transferred to rooting medium and grown for 4–5 weeks under the same conditions in Magenta jars. Single root-bearing plantlets were transferred onto a peat moss-based soil-less mixture [42] in 15 cm pots and covered with clear plastic cups for the first few days while acclimating to greenhouse conditions (16 h photoperiod; 22/18 °C; 230 μE.m^−2^.s-^1^). After acclimation, plants were uncovered and allowed to grow in the same greenhouse until maturity. At bolting, plants were encased in perforated pollination bags (Cryovac Sealed Air, Canada) and gently shaken manually once every two days to promote self-pollination. Mature T_1_ seeds were collected into paper envelopes, stored at room temperature, then used for developing seed lines. under the same greenhouse conditions. Longer term seed storage was at 4 °C.

### Transgenic *B. napus* selection/confirmation

O-TTG1 and K-TTG1 T_0_*B. napus* plants were confirmed by PCR of young leaf gDNA (DNeasy^®^ Plant Mini Kit) using *BAR* gene-specific primers (FBAR, RBAR: Additional file 2: Table S1). *GUS intron*-specific primers (FGUS, RGUS: Additional file 2: Table S1A) were used to further confirm K-TTG1 (RNAi) knockdown transformants (Additional file 1: Figure S2). The number of independent insertion loci was determined in T_0_ or T_1_ plants by Southern hybridization using a random-primed purified ^32^P-labelled 438 bp specific fragment of the PPT-resistant *BAR* gene (amplified from p79-103) or the 1,929 bp *AtGL3*gene as probes. Leaf gDNA (20 μg) was digested with 40 U HindIII (to avoid cutting within the BAR gene) or Xba1 or Kpn1 (to analyze *AtGL3*), separated on a 1 % agarose gel, and transferred overnight in 0.4 M NaOH to Amersham Hybond™-N^+^ nylon membranes (GE Healthcare). Cross-linked membranes were hybridized with probe overnight at 65 °C in SSC, 1 % BSA, 7 % SDS, 1 mM EDTA, and 0.5 M NaHPO4, pH 7.2 then washed twice each with 2x SSC-0.1 % SDS for 15 min at 65 °C and 0.2x SSC-0.1 % SDS for 15 min each at 65 °C. Autoradiography of the membranes was conducted using Kodak scientific imaging film (BioMax XAR) at −80 °C.

Introduction of the O-TTG1 (over-expression) construct into glabrous *B. napus* cv Westar resulted in 12 confirmed transformants out of 500 explants, while transformants with K-TTG1 (RNAi knock-down) resulted in seven confirmed transformants (Additional file 1: Figure S2, Table 1), This represented a 40-65 % “leakage” (false positive) in the Westar background using 5 mg/L L-phosphinotricin for selection. Screening transformants (using the same PPT concentration) harboring these constructs in the AtGL3^+^*B. napus* background gave seven out of nine PPT-tolerant plants that were positive for O-TTG1 and nine of 10 that were positive for the K-TTG1 RNAi construct. (Additional file 1: Figure S2, Table 1). Of the T_0_ plants confirmed by Southern analysis as K-TTG1-positive in the AtGL3^+^*B. napus* background, 50 % had a single transgene locus whereas only 25 % (two) of O-TTG1 positive plants in the AtGL3^+^*B. napus* background had a single transgene locus (Additional file 1: Figure S3A; Table 1). Of three T_0_ positive K-TTG1 transformants in the *B. napus* Westar background, only two lines (K-4 and K-16) had single transgene loci, while five independent single O-TTG1 insertions in the Westar background were identified (Additional file 1: Figure S3B,C; Table 1). Two lines K-5-8 and O-3-7 to be advanced out of the AtGL3^+^*B. napus* transformants were confirmed in subsequent Southern blot experiments to harbor a single insertion locus for *AtGL3* (Additional file 1: Figure S3D).

### Advancement of plants to form homozygous transgenic lines

Q-PCR relative expression of *BnTTG1* was used as a second screen with leaf tissues of greenhouse-grown T_0_ plantlets to select plants for advancement into homozygous lines. Q-PCR selection primers (FBAR, RBAR, FGUS, and RGUS) were designed and synthesized as above (detailed in Additional file 2: Table S1A) and Q-PCR analysis conducted as described below for batch leaf analysis. Six of seven positive K-TTG1 transformants in Westar showed reduced transcript levels for *BnTTG1* relative to Westar (Additional file 1: Figure S4A). Plant line K-13 showed the lowest expression (10-fold reduction), while the two single insert locus lines, K-4 and K-16, showed 5-fold and 0.75-fold lower relative expression levels relative to that of the four insert loci K-10 line. Relative expression of *BnTTG1* was also measured in 11 positive O-TTG1 T_0_ transformants in Westar (Additional file 1: Figure S4C). Eight showed increased *BnTTG1* expression, with highest expression (~70-fold increase) observed in the single insert locus plant O-12. Single insert locus plants O-2, O-9 and O-11 all showed expression levels similar to that of Westar.

Of the four single insert locus K-TTG1 plants in the AtGL3^+^*B. napus* background, K-5 and K-6 showed a 20-fold and 10-fold reduction in *BnTTG1*, respectively. They also showed an enhanced trichome coverage compared to hairy AtGL3^+^*B. napus* plants (Additional file 1: Figure S4B). Hence, these two K plants were carried through to form the homozygous lines K-5-8 and K-6-3. Plants K-8 and K-10 also showed reduced *BnTTG1* expression, but to a lesser degree than that of the K-5 and K-6 lines (Additional file 1: Figure S4B), and their trichome phenotypes were no different from the AtGL3^+^*B. napus* background line. Expression of *BnTTG1* in the glabrous T_0_ O-TTG1 over-expression plants in the AtGL3^+^*B. napus* background was not measured in this expression screen, since only one T_0_ plant (0–3) set seed and could be carried through to produce the homozygous line 0-3-7.

### TDNA insert positions

Two single-insert locus K-TTG1 lines in the AtGL3^+^*B. napus* background (K-5-8 and K-6-3) were analyzed using a Genome Walker^TM^ Universal Kit (Clontech) with separate DraI and EcoRV digestions to develop two GW libraries per line. BAR gene-specific primers (GW-BAR, GW-BAR’: Additional file 2: Table S1A) were used to generate PCR products for each library, and 10 colonies for each PCR product were sequenced to determine the position of the T-DNA loci in the *B. napus* lines. In the K-5-8 line, insertion occurred in the intergenic region ~2 kb downstream of the *B. napus* orthologue of the Arabidopsis *BOI-RELATED GENE2* (*BRG2*) (At1g79110, E value: 4e-59) involved in resistance to *Botrytis cinerea*. The T-DNA insertion in K-6-3 was found within the *B. napus* orthologue of Arabidopsis *SKU5 SIMILAR 2* (*SKS2*) (At5g51480, E value: 2e-68), the protein product of which locates to the plasma membrane and functions in oxido-reductase activity and copper ion binding (Additional file 1: Figure S5A). None of these SALK knock-out lines for the Arabidopsis orthologues of these two genes (i.e., SALK_070108C; SALK_127570; SALK_074628; SALK_070108C; SALK_074627; SALK_127112C; SALK_070255 and SALK_036510) showed any difference in growth or trichome morphology when compared to the wild type Columbia phenotype (Additional file 1: Figure S5). Since these genes are most likely duplicated in *B. napus*, their disrupted single knockout loci in *B. napus* likely are not involved in changes to the transgenic *B. napus* trichome and growth phenotypes, nor to the extreme changes noted in the transcriptomes of these novel plants.

### Greenhouse growth measurements

Homozygous *B. napus* plant lines (ultra-hairy leaf K-5-8, ultra-hairy leaf K-6-3, glabrous leaf T_3_ O-3-7, hairy leaf AtGL3^+^*B. napus*, and semi-glabrous leaf cv. Westar) were cultivated in 15 cm pots and grown at the same time in a controlled environment greenhouse (AAFC-SRC) (and once in a growth cabinet) under previously described conditions to examine their phenotypes. Side-by-side growth was evaluated four times: once including the K-6-3 line, but without the glabrous T_3_ O-3-7 seed line (which took more than 2 years longer to recover than the K-5-8 and K-6-3 lines); once including the glabrous T_3_ O-3-7 line but without the K-6-3 line; and twice 1.5 years later without the K-6-3 line, but evaluating growth and trichome phenotypes in two independently generated O-3-7 seed lots (T_4_ O-3-7-5 and T_4_ O-3-7-3 plants) in both greenhouse pots and in Magenta jars in a growth cabinet. Pots were covered with clear plastic lids until seeds had germinated and small plantlets had emerged. Seedling emergence was documented until all seeds were germinated (up to 10 days after seeding). Plant height, trichome and growth phenotypes, and seed yield were recorded for each plant. Empty vector control plants were also phenotyped visually for growth and trichome phenotypes. When plants started flowering, they were wrapped individually with perforated pollination bags and gently shaken by hand once every two days to promote self-pollination. Mature seed pods were collected into paper bags and seed was stored at 4 °C.

### Microscopy of trichome bearing tissues

Visible trichome phenotypes were recorded on all O-TTG1 and K-TTG1 plants during line establishment and stability testing, and T_3_/T_4_ O-3-7, K-5-8, AtGL3^+^*B. napus*, and Westar homozyous lines for experimental purposes, grown under controlled greenhouse conditions (above) using a Panasonic, Lumix (DMC-FZ50) camera. For detailed trichome analysis, small leaf disks were punched from the first six leaves of wild-type (semi-glabrous cv. Westar) and hairy and ultra-hairy transgenic *B. napus* lines, then directly mounted on aluminum stubs (9 mm dia. disk limit). Observations on trichome density and structure were made using a Phillips 505 (30 kV) scanning electron microscope (Department of Biology, University of Saskatchewan), since trichome densities on both hairy and ultra-hairy lines were too high for accurate assessment with a lower resolution microscope. Electron microscopy had been successfully used earlier to measure very high trichome density in *B. villosa* [35]. Micrograph images maximally covering <2 mm^2^ were photographed on FP-100B panchromatic type Polaroid film (Fujifilm, Tokyo, Japan), and numbers of trichomes were counted on digital micrograph images of a 1 mm^2^ area of the adaxial leaf surface on six leaves per plant (three separate positions per leaf) for three independent plants per line and adjusted to represent trichome density in a 1 cm^2^ area. This enabled a consistent, statistically significant, accurate comparison of trichome density between lines on the small images. For the petiole, leaf edge and abaxial mid-vein surface, number of trichomes on a 1 mm length were counted and adjusted to represent density per cm length. To measure trichome length, trichomes were collected from the first to the fourth leaf of 65 plants per line by vortexing 3-week-old seedlings in a 50 mL tube containing liquid N_2_ according to [34]. Recovered trichome preparations were viewed under a ZEISS Axio Vision epiflorescent microscope, and lengths were measured using an Axio Vision Documentation system (ZEISS, Jena, Germany).

### Quantitative RT-PCR (Q-PCR) using summarized expression of trichome genes and leaf batches combining different development stages

RNA was extracted using the commercial RNeasy^®^ Mini Kit (Qiagen) from leaf tissue of cv Westar, homozygous hairy and ultra-hairy *B. napus* plants (AtGL3^+^*B. napus* and K-5-8, respectively), and homozygous glabrous T_3_ O-3-7) grown in soil in a growth chamber under 22/18 °C (day/night) and 16/8 h (light/dark) (400 μE.m^−2^.s^−1^). The first three leaves (from five 16 day-old seedlings with the third leaf still unfolding) were harvested and combined into one tissue batch, while the fourth, fifth, and sixth leaves (from five 23-day-old seedlings with the sixth leaf still unfolding) were combined into a second batch, to make one rep per line. First-strand cDNA synthesis was performed using 5 μg RNA, SuperScript™ II reverse transcriptase (Invitrogen), and Oligo (dT)12-18 primers. Final Q-PCR mixtures contained 1x Platinum^®^ SYBR^®^ Green qPCR SuperMix-UDG (Invitrogen), 10 μM of each gene-specific forward and reverse primer (Additional file 2: Table S1), and 100 ng cDNA to a final volume of 20 μL. Gene-specific primers for six commonly known trichome regulatory genes involved with the MBW activator complex [2] and the two housekeeping/control genes were designed (as below) based on available sequence data at the time of experimentation (details on primer sequences in Additional file 2: Table S1A). Q-PCR gene fragments were amplified using a CFX96 Manager Real-Time thermal cycler (Bio-Rad, Hercules, CA, USA) for 50 °C/2 min, 95 °C/2 min, 40 x [95 °C/15 s, 60 °C/30 s], 95 °C/10 s, followed by a melting curve and 4 °C/∞. All Q-PCR experiments included the *B. napus ACYL TRANSFERASE 2* (*ACT2*) gene (Accession # AF111161.1, www.ncbi.nlm.nih.gov) as the Q-PCR control gene in all combined tissue samples. Expression of *BnACT2* and the “housekeeping” gene *ELONGATION FACTOR 1* (*BnEF1*) (Accession # FJ529180, www.ncbi.nlm.nih.gov) were pre-tested for expression stability relative to each other in several tissues. “Summarized” gene expression data for combined orthologues and paralogues of each trichome regulatory gene (detailed below) was then reported relative to *BnACT2* expression in cotyledons of each individual line (not shown; set at 1), since both *BnACT2* and *BnEF1* showed stable expression in cotyledons and leaf batches of all four tested lines. This allowed for comparison of expression of trichome genes across the multiple Q-PCR plates required for this experiment.

Since the *B. napus* genome was not available at the time of this particular Q-PCR experiment (and hence individual gene orthologues and paralogues were not necessarily known), primer pairs for the five trichome regulatory genes were designed from full length cDNA sequences retrieved from NCBI (www.ncbi.nlm.nih.gov) in conserved regions that would likely “capture” all copies expressed in *B. napus* leaves (Additional file 2: Table S1A) to give a combined “summarized” leaf expression level. For *GL1*, we used a conserved region between BN_GL1 - HQ162473 (*B. napus*), BI_GL1 - HQ162472 (*B. incana*), BO_GL1 - HQ162471 (*B. oleracea*) and BR_GL1 - HQ162470 (*B. rapa*). For *GL2*, we used a conserved region between EU826520 - (*BnGL2a*) and EU826521 (*BnGL2b*). For *TTG1*, alignments were generated between EF175930 (*B. napus* isoform 1), EF175932 (*B. napus* isoform 2), and HM208590.1 (*B. rapa*), and for *TRY*, a conserved region was found between an EST sequence for *BnTRY* (EE451172 from http://brassicadb.org/brad/) and the Arabidopsis AtTRY (AT5G53200**)** (Additional file 2: Table S1A), However, no *BnGL3* sequences were available at the time. Hence, Arabidopsis *AtGL3* cDNA was exhaustively screened with multiple primer pairs, only one of which amplified a *BnGL3* product (~500 bp) (Accession # Pr032754350, www.ncbi.nlm.nih.gov). *B. napus* Q-PCR primers were then designed to a variable region that distinguished the *BnGL3* sequence from the *AtGL3* and *BnEGL3* sequences (Additional file 2: Table S1A). A recent BLAST analysis of each primer pair against the *B. rapa, B. oleracea,* and *B. napus* databases in NCBI, followed by a search for *BnGL3* sequences missing from these databases and manual alignment of the primers confirmed mainly 100 % gene identity for these primers (with very few exceptions) (Additional file 2: Table S1B,C). [Unfortunately, the server housing a new *B. oleracea* genome (Parkin) was unavailable at the time to confirm the copy numbers retrieved from these other databases.] These results were then cross-referenced to the different *B. rapa-*related and *B. oleracea*-related trichome orthologues expressed in *B. napus* first leaves as measured by RNA sequencing (below) to determine how many trichome regulatory gene copies were likely represented in the batch leaf samples (Additional file 2: Table S1C, derived from Additional file 2: Tables S2A and S2B).

### RNA sequencing of first leaves from Westar, AtGL3^+^*B. napus*, and K-5-8

Seeds from semi-glabrous leaf Westar, hairy leaf AtGL3^+^*B. napus*, and ultra-hairy leaf K-5-8 were germinated in three replicates (10 seeds/replicate) and plantlets grown in Magenta jars in a controlled growth chamber (as described above). At the time of leaf sampling for RNA sequencing, the glabrous leaf T_3_ O-3-7 line was not available due to low fertility of plants containing the O-TTG1 construct. Total RNA was extracted from unfolded first leaves before emergence of the second leaf (10-day-old seedlings). RNA was extracted using an RNAeasy Mini Kit with contaminating gDNA removed by RNAse-free DNAse™ (Qiagene Inc., ON). RNA samples were quantified and RNA integrity determined using a RNA6000 nano assay in the Agilent 2100 Bioanalyzer™ (Agilent Technologies, Palo Alto, CA). RNA library preparation and sequencing was carried out at the National Research Council (Saskatoon, Canada) using the Illumina TrueSeq RNA sample preparation platform v.2 with multiplex labeling following the manufacturer’s protocols. cDNA libraries were validated using the 2100 Bioanalyzer™ and quantified by Q-PCR. Multiplexed samples (22 samples per lane) were sequenced in paired-end reads with 101 cycles. Initial base calling and quality filtering of Illumina HiSeq 2000 image data was performed using the default parameters of the Illumina HiSeq2000 Pipeline GERALD stage. A quality check was performed on the raw sequencing data with FastQC (http://www.bioinformatics.babraham.ac.uk/projects/fastqc/). Trimmomatic 0.20 was used to remove adaptor sequences and to trim bases with quality lower than 20 (phred 33 quality scores) on the first 12 nucleotides of each read according to Illumina’s recommendations [43], raw RNA-SEQ data was submitted to www.ncbi.nlm.nih.gov and accession number for the submission is SRP065063.

In the absence of a *B. napus* reference genome, the processed sequencing reads (ESTs) were aligned to a synthetic reference genome, created by combining sequenced annotated genomes from *B. rapa* [39] and *B. oleracea* [40]. EST read mapping was carried out using TopHat v.2.0.7 [44]. Read counting and extraction of FPKM values (Fragment Per Kilobase of exon per Million fragments mapped) was carried out using the Cufflinks tool suite [44]. To compare experimental transcripts to predicted transcripts from the synthetic *B. napus* genome, merged gtf files for transcripts identified by the Toxedo package (providing the assembled transcripts) were compared to the individual *B. rapa* and in-house *B.oleracea* gtf annotation files provided by BrassicaDB.org [40] and I. Parkin [41], respectively. Analysis and visualization of the RNAseq data was performed in R with a cummeRbund package [44]. Since gene expression changes 1.5- to 2-fold relative to cv. Westar leaf expression had been found to be meaningful in an earlier comparison of transcripts from extremely hairy leaves of *B. villosa* using microarray analysis [34], genes with this level of relative expression or greater were categorized into 35 sub-cellular function categories or sub-categories using Mapman [45]. Trichome genes (category 36) were retrieved through searches of literature databases and TAIR as specified in [8] and categorized into function sub-categories based on development criteria.

### Q-PCR analysis on individual *B. napus* genes specifying redox, cell wall carbohydrate, lignin, and trichome pathways in first leaves

Semi-glabrous leaf Westar, hairy leaf AtGL3^+^*B. napus*, ultra-hairy leaf K-5-8 and T_4_ O-3-7 (showing partial reversion to a moderately hairy leaf phenotpe) were grown under conditions described for RNA sequencing, and total RNA was extracted from young seedling first leaves. Only T_4_ O-3-7 plants showing the moderately hairy leaf phenotype and the taller healthier growth phenotype (like the AtGL3^+^*B. napus* line) were selected for RNA extraction, since the smaller T_4_ phenotype died before sufficient first tissue could be collected. cDNA was synthesized and Q-PCR assays conducted on three independent preparations per species as described above for batch leaf Q-PCR, with the exception of a diffrent endogenous reference gene. Genes specifying redox, cell wall carbohydrate, lignin, and trichome genes were selected on the basis of RNA-sequencing results for K-5-8 and the AtGL3^+^*B. napus* lines (Additional file 2: Tables S2A, 2B, S4, S10, and S15). Since *B. napus* has one copy of the *B. rapa* genome and one copy of the *B.oleracea* genome, Q-PCR primer pairs were designed using unique regions differing between *B. rapa* and *B. oleracea* genes in NCBI wherever unique orthologous sequence was available; otherwise primer pairs were designed within regions conserved with homologues from other species (Additional file 2: Table S1A). For each pair of specific primers, melting curve analysis was conducted to determine melt temperature and ensure a single PCR amplicon of the expected length (Additional file 2: Table S1A). The expression level of each mRNA was determined using the mean cycle threshold (ΔCT) value normalized to an endogenous *B. napus* reference gene, *Glyceraldehyde-3-phosphate-dehydrogenase* (*BnGADH2*) (Additional file 2: Table S1A) and mean values with corresponding standard errors expressed relative to glabrous *B. napus* leaf tissue (set at 1).

### Statistical analysis

Data for growth, trichome measurements, and Q-PCR were analyzed with either one-way or two-way ANOVA using a MIXED model in SAS 9.2 [46]. Assumptions of ANOVA were tested using a Normality test and Levenes test. Means were compared using a Tukey test in SAS 9.2, and treatments were declared significant at *P* ≤ 0.05 and trends declared at *P* ≤ 0.1. Read counting and statistical analysis of the RNA-seq data were carried out using Cuffdiff in the Cufflinks software package 44].

## Availability of supporting data

Raw RNA-SEQ data was submitted to www.ncbi.nlm.nih.gov under the accession number SRP065063.

## Plant Line Abbreviations (genotypes; phenotypes)

**K-5-8** (*35S*_*p*_*::AtGL3* expression together with 35S_p_::*BnTTG1* RNAi knockdown in *B. napus*; stable phenotype with ultra-hairy leaves and fully restored growth).

**0-3-7** (*35S*_*p*_*::AtGL3* expression together with *35S*_*p*_*::BnTTG1* over-expression in *B. napus*; low vitality T_3_ plants with glabrous leaves and stems, poor seed quality, and smaller size than Westar. Instability showed with the emergence of two new phenotypes in T_4_ plants: somewhat smaller plants than Westar with intermediate trichome density, and very small plants with trichome density somewhat higher than Westar).

**GL3+*B. napus*** (*35S*_*p*_*::AtGL3* expression in *B. napus* Westar; stable phenotype with hairy leaves, but somewhat smaller plants with later maturity than Westar).

**Westar** (*B. napus* cultivar Westar; semi-glabrous leaves and good growth).

## Additional files

Additional file 1: Figure S1. Alignments between *B. napus* and *B. rapa TTG1* sequences, primer coverage, and binary vector construction for manipulating *TTG1* in *B. napus. B. napus TTG1-1* (EF175930), *TTG1-2* (EF175932) and *B. rapa TTG1* (HM208590.1). **Figure S2.** PCR confirmation of O-TTG1 or K-TTG1 TDNA in putative phosphinothricin tolerant T_0_ transformants of *B. napus* cv Westar and AtGL3^+^ *B. napus*. **Figure S3.** Representative Southern analysis to detect copy number of K-TTG1 and O-TTG1 TDNA in AtGL3^+^ *B. napus* and Westar. **Figure S4.** Relative expression of *BnTTG1* in seedling tissues of T_0_ plants transformed with K-TTG1 or O-TTG1 binary vectors in semi-glabrous cv. Westar and hairy AtGL3^+^ *B. napus* backgrounds using Q-PCR. **Figure S5.** Fourth rosette leaf trichome phenotypes of two-week-old *Arabidopsis thaliana* SALK lines 074628 (*BRG2*), 127112C (*BRG2*) and 036510 (*SKS2*). **Figure S6.** Stem trichomes on T_0_ hairy *B. napus* K-5-8 at eight weeks after germination. **Figure S7.** Heatmap illustrating the relative expression levels and numbers of unique up-regulated and down-regulated genes (*p* < 0.05) in ultra-hairy K-5-8 leaves and hairy AtGL3^+^ *B. napus* in a range of MapMan functional sub-categories (relative to semi-glabrous Westar leaves). **Figure S8.** Amino acid alignment between Arabidopsis AtGL3 and BnGL3 homologues in *B. napus.* **Figure S9.** Developmental expression profile of *AtTTG1* and *AtGL3* using *in silico* microarray data on the Arabidopsis eFP and TileViz browsers. (PPTX 4419 kb) Additional file 2: Table S1A. Primer sequences used in the construction and analysis of *B. napus* transgenic lines. **Table S1B.** Blast of batch leaf Q-PCR primers to the *B. rapa*, *B. oleracea*, and *B. napus* genomes for five trichome regulatory genes and two control genes in *B. napus*. **Table S1C.** “Detectable” *B. napus* homologues of five trichome regulatory genes in first true leaves (from RNA sequencing). **Table S1D.** BlastP for five Arabidopsis trichome regulatory genes against the *Brassica napus* genome in NCBI. **Table S2A.** Differentially expressed leaf trichome ESTs (*p* < 0.05) in hairy AtGL3^+^ *B. napus* or ultra-hairy line K-5-8 relative to semi-glabrous cv. Westar. **Table S2B.** Leaf trichome genes with no significant expression differences (*p* < 0.05) in hairy AtGL3^+^ *B. napus* or ultra-hairy line K-5-8 relative to semi-glabrous cv. Westar. **Table S3.** Differentially expressed leaf flavonoid ESTs (*p* < 0.05) in hairy AtGL3^+^ *B. napus* or ultra-hairy K-5-8 relative to semi-glabrous cv. Westar. **Table S4.** Differentially expressed leaf phenylpropanoid and lignin ESTs (*p* < 0.05) in hairy AtGL3^+^ *B. napus* or ultra-hairy K-5-8 relative to semi-glabrous cv. Westar. **Table S5.** Differentially expressed leaf phenolic ESTs (*p* < 0.05) in hairy AtGL3^+^ *B. napus* or ultra-hairy K-5-8 relative to semi-glabrous cv. Westar. **Table S6.** Differentially expressed leaf shikimate ESTs (*p* < 0.05) in hairy AtGL3^+^ *B. napus* or ultra-hairy K-5-8 relative to semi-glabrous cv. Westar. **Table S7.** Differentially expressed leaf isoprenoid and terpene ESTs (*p* < 0.05) in hairy AtGL3^+^ *B. napus* or ultra-hairy K-5-8 relative to semi-glabrous cv. Westar. **Table S8.** Differentially expressed leaf glucosinolate-related and miscellaneous sulphur-related ESTs (*p* < 0.05) in hairy AtGL3^+^ *B. napus* or ultra-hairy K-5-8 relative to semi-glabrous cv. Westar. **Table S9.** Differentially expressed leaf alkaloid-related and miscellaneous N-metabolizing ESTs (*p* < 0.05) in hairy AtGL3^+^ *B. napus* or ultra-hairy K-5-8 relative to semi-glabrous cv. Westar. **Table S10.** Differentially expressed leaf cell wall structural carbohydrate ESTs ((*p* < 0.05) in hairy AtGL3^+^ *B. napus* or ultra-hairy K-5-8 relative to semi-glabrous cv. Westar. **Table S11.** Differentially expressed leaf mucilage ESTs (*p* < 0.05) in hairy AtGL3^+^ *B. napus* or ultra-hairy K-5-8 relative to semi-glabrous cv. Westar. **Table S12.** Differentially expressed leaf wax ESTs (*p* < 0.05) in hairy AtGL3^+^ *B. napus* or ultra-hairy K-5-8 relative to semi-glabrous cv. Westar. **Table S13.** Differentially expressed leaf hormone ESTs (*p* < 0.05) in hairy AtGL3^+^ *B. napus* or ultra-hairy K-5-8 relative to semi-glabrous cv. Westar. **Table S14.** Differentially expressed leaf secondary metabolism ESTs (*p* < 0.05) in hairy AtGL3^+^ *B. napus* or ultra-hairy K-5-8 relative to semi-glabrous cv. Westar. **Table S15.** Differentially expressed leaf redox-related ESTs (*p* < 0.05)) in hairy AtGL3^+^ *B. napus* or ultra-hairy K-5-8 relative to semi-glabrous cv. Westar. **Table S16.** Differentially expressed leaf protein modification ESTs (*p* < 0.05) in hairy AtGL3^+^ *B. napus* or ultra-hairy K-5-8 relative to semi-glabrous cv. Westar. **Table S17.** Differentially expressed leaf protein degradation ESTs (*p* < 0.05) in hairy AtGL3^+^ *B. napus* or ultra-hairy K-5-8 relative to semi-glabrous cv. Westar. **Table S18.** Differentially expressed leaf transcription factor ESTs (*p* < 0.05) in hairy AtGL3^+^ *B. napus* or ultra-hairy K-5-8 relative to semi-glabrous cv. Westar. (XLSX 400 kb)

## Abbreviations

RNA: ribonucleic acid
DNA: deoxy ribonucleic acid
Q-PCR: quantitative polymerase chain reaction
DEG: differentially expressed gene
EST: expressed sequence tag
RNA-SEQ: ribonuleic acid sequencing
T-DNA: transfer deoxy ribonucleic acid
