# Supplementary material for: Hairy Canola (Brasssica napus) re-visited: Down-regulating TTG1 in an AtGL3-enhanced hairy leaf background improves growth, leaf trichome coverage, and metabolite gene expression diversity
Source: BMC Plant Biol. 2016 Jan 6;16:12. doi: 10.1186/s12870-015-0680-5 (PMC4704247; doi:10.1186/s12870-015-0680-5)
Supplement: Additional file 1: Figure S1. — Alignments between B. napus and B. rapa TTG1 sequences, primer coverage, and binary vector construction for manipulating TTG1 in B. napus. B. napus TTG1-1 (EF175930), TTG1-2 (EF175932) and B. rapa TTG1 (HM208590.1). Figure S2. PCR confirmation of O-TTG1 or K-TTG1 TDNA in putative phosphinothricin tolerant T0 transformants of B. napus cv Westar and AtGL3+ B. napus. Figure S3. Representative Southern analysis to detect copy number of K-TTG1 and O-TTG1 TDNA in AtGL3+ B. napus and Westar. Figure S4. Relative expression of BnTTG1 in seedling tissues of T0 plants transformed with K-TTG1 or O-TTG1 binary vectors in semi-glabrous cv. Westar and hairy AtGL3+ B. napus backgrounds using Q-PCR. Figure S5. Fourth rosette leaf trichome phenotypes of two-week-old Arabidopsis thaliana SALK lines 074628 (BRG2), 127112C (BRG2) and 036510 (SKS2). Figure S6. Stem trichomes on T0 hairy B. napus K-5-8 at eight weeks after germination. Figure S7. Heatmap illustrating the relative expression levels and numbers of unique up-regulated and down-regulated genes (p < 0.05) in ultra-hairy K-5-8 leaves and hairy AtGL3+ B. napus in a range of MapMan functional sub-categories (relative to semi-glabrous Westar leaves). Figure S8. Amino acid alignment between Arabidopsis AtGL3 and BnGL3 homologues in B. napus. Figure S9. Developmental expression profile of AtTTG1 and AtGL3 using in silico microarray data on the Arabidopsis eFP and TileViz browsers. (PPTX 4419 kb) [file 12870_2015_680_MOESM1_ESM.pptx]

## Slide 1
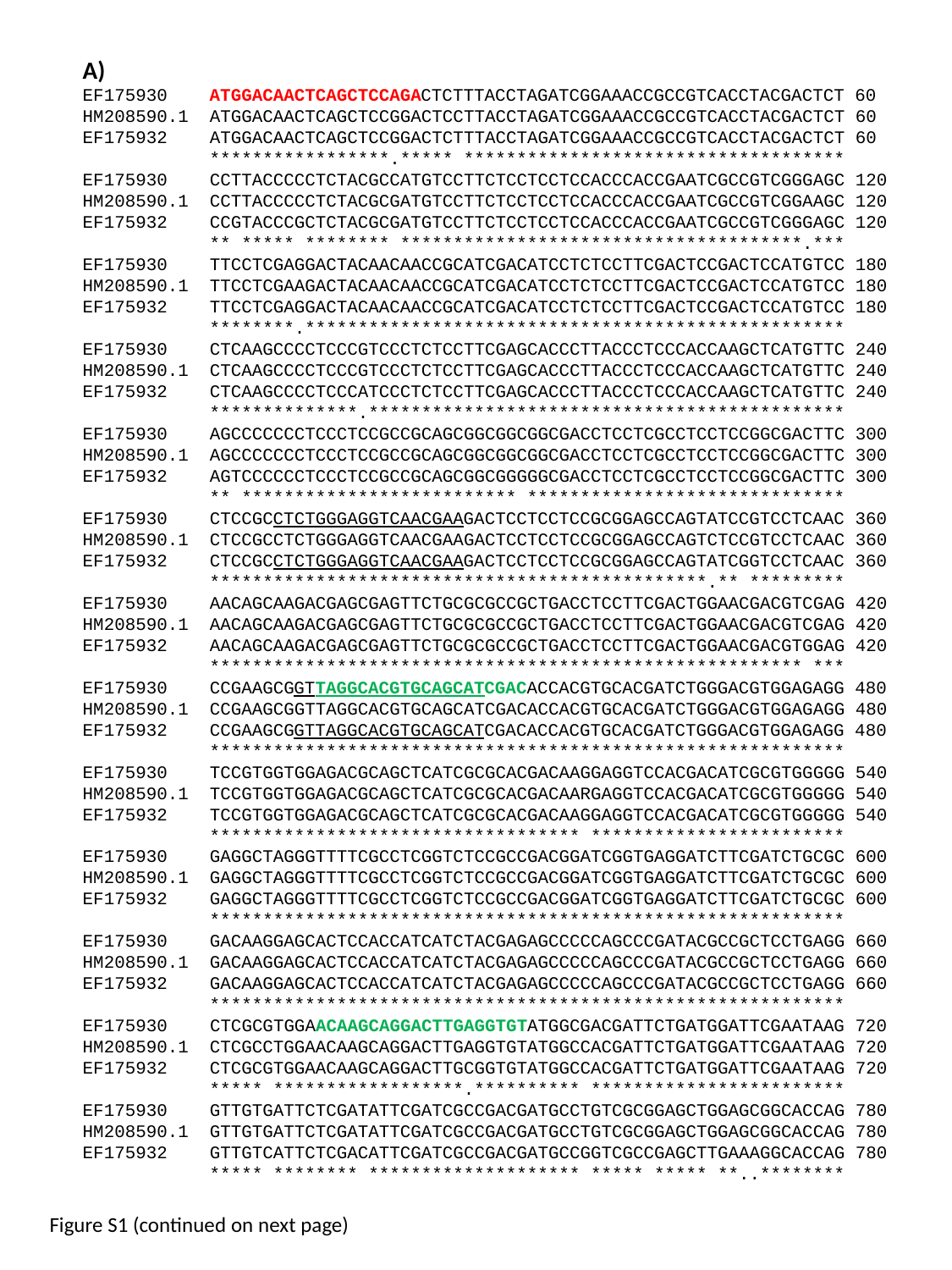

A)
EF175930 ATGGACAACTCAGCTCCAGACTCTTTACCTAGATCGGAAACCGCCGTCACCTACGACTCT 60
HM208590.1 ATGGACAACTCAGCTCCGGACTCCTTACCTAGATCGGAAACCGCCGTCACCTACGACTCT 60
EF175932 ATGGACAACTCAGCTCCGGACTCTTTACCTAGATCGGAAACCGCCGTCACCTACGACTCT 60
 *****************.***** ************************************
EF175930 CCTTACCCCCTCTACGCCATGTCCTTCTCCTCCTCCACCCACCGAATCGCCGTCGGGAGC 120
HM208590.1 CCTTACCCCCTCTACGCGATGTCCTTCTCCTCCTCCACCCACCGAATCGCCGTCGGAAGC 120
EF175932 CCGTACCCGCTCTACGCGATGTCCTTCTCCTCCTCCACCCACCGAATCGCCGTCGGGAGC 120
 ** ***** ******** **************************************.***
EF175930 TTCCTCGAGGACTACAACAACCGCATCGACATCCTCTCCTTCGACTCCGACTCCATGTCC 180
HM208590.1 TTCCTCGAAGACTACAACAACCGCATCGACATCCTCTCCTTCGACTCCGACTCCATGTCC 180
EF175932 TTCCTCGAGGACTACAACAACCGCATCGACATCCTCTCCTTCGACTCCGACTCCATGTCC 180
 ********.***************************************************
EF175930 CTCAAGCCCCTCCCGTCCCTCTCCTTCGAGCACCCTTACCCTCCCACCAAGCTCATGTTC 240
HM208590.1 CTCAAGCCCCTCCCGTCCCTCTCCTTCGAGCACCCTTACCCTCCCACCAAGCTCATGTTC 240
EF175932 CTCAAGCCCCTCCCATCCCTCTCCTTCGAGCACCCTTACCCTCCCACCAAGCTCATGTTC 240
 **************.*********************************************
EF175930 AGCCCCCCCTCCCTCCGCCGCAGCGGCGGCGGCGACCTCCTCGCCTCCTCCGGCGACTTC 300
HM208590.1 AGCCCCCCCTCCCTCCGCCGCAGCGGCGGCGGCGACCTCCTCGCCTCCTCCGGCGACTTC 300
EF175932 AGTCCCCCCTCCCTCCGCCGCAGCGGCGGGGGCGACCTCCTCGCCTCCTCCGGCGACTTC 300
 ** ************************** ******************************
EF175930 CTCCGCCTCTGGGAGGTCAACGAAGACTCCTCCTCCGCGGAGCCAGTATCCGTCCTCAAC 360
HM208590.1 CTCCGCCTCTGGGAGGTCAACGAAGACTCCTCCTCCGCGGAGCCAGTCTCCGTCCTCAAC 360
EF175932 CTCCGCCTCTGGGAGGTCAACGAAGACTCCTCCTCCGCGGAGCCAGTATCGGTCCTCAAC 360
 ***********************************************.** *********
EF175930 AACAGCAAGACGAGCGAGTTCTGCGCGCCGCTGACCTCCTTCGACTGGAACGACGTCGAG 420
HM208590.1 AACAGCAAGACGAGCGAGTTCTGCGCGCCGCTGACCTCCTTCGACTGGAACGACGTCGAG 420
EF175932 AACAGCAAGACGAGCGAGTTCTGCGCGCCGCTGACCTCCTTCGACTGGAACGACGTGGAG 420
 ******************************************************** ***
EF175930 CCGAAGCGGTTAGGCACGTGCAGCATCGACACCACGTGCACGATCTGGGACGTGGAGAGG 480
HM208590.1 CCGAAGCGGTTAGGCACGTGCAGCATCGACACCACGTGCACGATCTGGGACGTGGAGAGG 480
EF175932 CCGAAGCGGTTAGGCACGTGCAGCATCGACACCACGTGCACGATCTGGGACGTGGAGAGG 480
 ************************************************************
EF175930 TCCGTGGTGGAGACGCAGCTCATCGCGCACGACAAGGAGGTCCACGACATCGCGTGGGGG 540
HM208590.1 TCCGTGGTGGAGACGCAGCTCATCGCGCACGACAARGAGGTCCACGACATCGCGTGGGGG 540
EF175932 TCCGTGGTGGAGACGCAGCTCATCGCGCACGACAAGGAGGTCCACGACATCGCGTGGGGG 540
 *********************************** ************************
EF175930 GAGGCTAGGGTTTTCGCCTCGGTCTCCGCCGACGGATCGGTGAGGATCTTCGATCTGCGC 600
HM208590.1 GAGGCTAGGGTTTTCGCCTCGGTCTCCGCCGACGGATCGGTGAGGATCTTCGATCTGCGC 600
EF175932 GAGGCTAGGGTTTTCGCCTCGGTCTCCGCCGACGGATCGGTGAGGATCTTCGATCTGCGC 600
 ************************************************************
EF175930 GACAAGGAGCACTCCACCATCATCTACGAGAGCCCCCAGCCCGATACGCCGCTCCTGAGG 660
HM208590.1 GACAAGGAGCACTCCACCATCATCTACGAGAGCCCCCAGCCCGATACGCCGCTCCTGAGG 660
EF175932 GACAAGGAGCACTCCACCATCATCTACGAGAGCCCCCAGCCCGATACGCCGCTCCTGAGG 660
 ************************************************************
EF175930 CTCGCGTGGAACAAGCAGGACTTGAGGTGTATGGCGACGATTCTGATGGATTCGAATAAG 720
HM208590.1 CTCGCCTGGAACAAGCAGGACTTGAGGTGTATGGCCACGATTCTGATGGATTCGAATAAG 720
EF175932 CTCGCGTGGAACAAGCAGGACTTGCGGTGTATGGCCACGATTCTGATGGATTCGAATAAG 720
 ***** ******************.********** ************************
EF175930 GTTGTGATTCTCGATATTCGATCGCCGACGATGCCTGTCGCGGAGCTGGAGCGGCACCAG 780
HM208590.1 GTTGTGATTCTCGATATTCGATCGCCGACGATGCCTGTCGCGGAGCTGGAGCGGCACCAG 780
EF175932 GTTGTCATTCTCGACATTCGATCGCCGACGATGCCGGTCGCCGAGCTTGAAAGGCACCAG 780
 ***** ******** ******************** ***** ***** **..********
Figure S1 (continued on next page)

## Slide 2
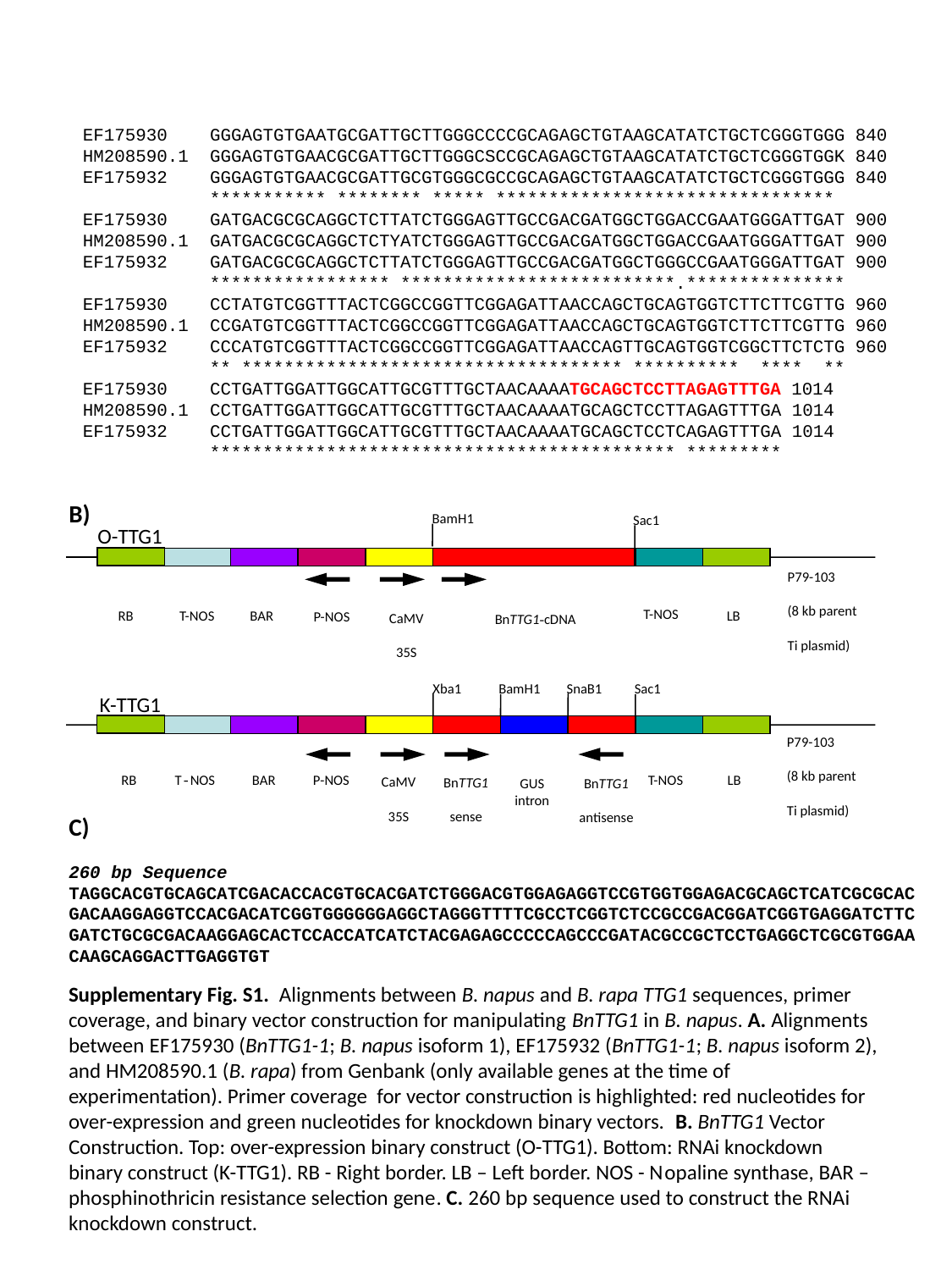

EF175930 GGGAGTGTGAATGCGATTGCTTGGGCCCCGCAGAGCTGTAAGCATATCTGCTCGGGTGGG 840
HM208590.1 GGGAGTGTGAACGCGATTGCTTGGGCSCCGCAGAGCTGTAAGCATATCTGCTCGGGTGGK 840
EF175932 GGGAGTGTGAACGCGATTGCGTGGGCGCCGCAGAGCTGTAAGCATATCTGCTCGGGTGGG 840
 *********** ******** ***** ********************************
EF175930 GATGACGCGCAGGCTCTTATCTGGGAGTTGCCGACGATGGCTGGACCGAATGGGATTGAT 900
HM208590.1 GATGACGCGCAGGCTCTYATCTGGGAGTTGCCGACGATGGCTGGACCGAATGGGATTGAT 900
EF175932 GATGACGCGCAGGCTCTTATCTGGGAGTTGCCGACGATGGCTGGGCCGAATGGGATTGAT 900
 ***************** **************************.***************
EF175930 CCTATGTCGGTTTACTCGGCCGGTTCGGAGATTAACCAGCTGCAGTGGTCTTCTTCGTTG 960
HM208590.1 CCGATGTCGGTTTACTCGGCCGGTTCGGAGATTAACCAGCTGCAGTGGTCTTCTTCGTTG 960
EF175932 CCCATGTCGGTTTACTCGGCCGGTTCGGAGATTAACCAGTTGCAGTGGTCGGCTTCTCTG 960
 ** ************************************ ********** **** **
EF175930 CCTGATTGGATTGGCATTGCGTTTGCTAACAAAATGCAGCTCCTTAGAGTTTGA 1014
HM208590.1 CCTGATTGGATTGGCATTGCGTTTGCTAACAAAATGCAGCTCCTTAGAGTTTGA 1014
EF175932 CCTGATTGGATTGGCATTGCGTTTGCTAACAAAATGCAGCTCCTCAGAGTTTGA 1014
 ******************************************** *********
B)
BamH1
Sac1
P79-103
(8 kb parent
Ti plasmid)
T-NOS
RB
BAR
LB
T-NOS
P-NOS
CaMV
35S
BnTTG1-cDNA
O-TTG1
Xba1
BamH1
SnaB1
Sac1
P79-103
(8 kb parent
Ti plasmid)
RB
T-NOS
BAR
P-NOS
T-NOS
LB
CaMV
35S
BnTTG1
sense
GUS intron
BnTTG1
antisense
K-TTG1
C)
260 bp Sequence
TAGGCACGTGCAGCATCGACACCACGTGCACGATCTGGGACGTGGAGAGGTCCGTGGTGGAGACGCAGCTCATCGCGCACGACAAGGAGGTCCACGACATCGGTGGGGGGAGGCTAGGGTTTTCGCCTCGGTCTCCGCCGACGGATCGGTGAGGATCTTCGATCTGCGCGACAAGGAGCACTCCACCATCATCTACGAGAGCCCCCAGCCCGATACGCCGCTCCTGAGGCTCGCGTGGAACAAGCAGGACTTGAGGTGT
Supplementary Fig. S1. Alignments between B. napus and B. rapa TTG1 sequences, primer coverage, and binary vector construction for manipulating BnTTG1 in B. napus. A. Alignments between EF175930 (BnTTG1-1; B. napus isoform 1), EF175932 (BnTTG1-1; B. napus isoform 2), and HM208590.1 (B. rapa) from Genbank (only available genes at the time of experimentation). Primer coverage for vector construction is highlighted: red nucleotides for over-expression and green nucleotides for knockdown binary vectors. B. BnTTG1 Vector Construction. Top: over-expression binary construct (O-TTG1). Bottom: RNAi knockdown binary construct (K-TTG1). RB - Right border. LB – Left border. NOS - Nopaline synthase, BAR – phosphinothricin resistance selection gene. C. 260 bp sequence used to construct the RNAi knockdown construct.

## Slide 3
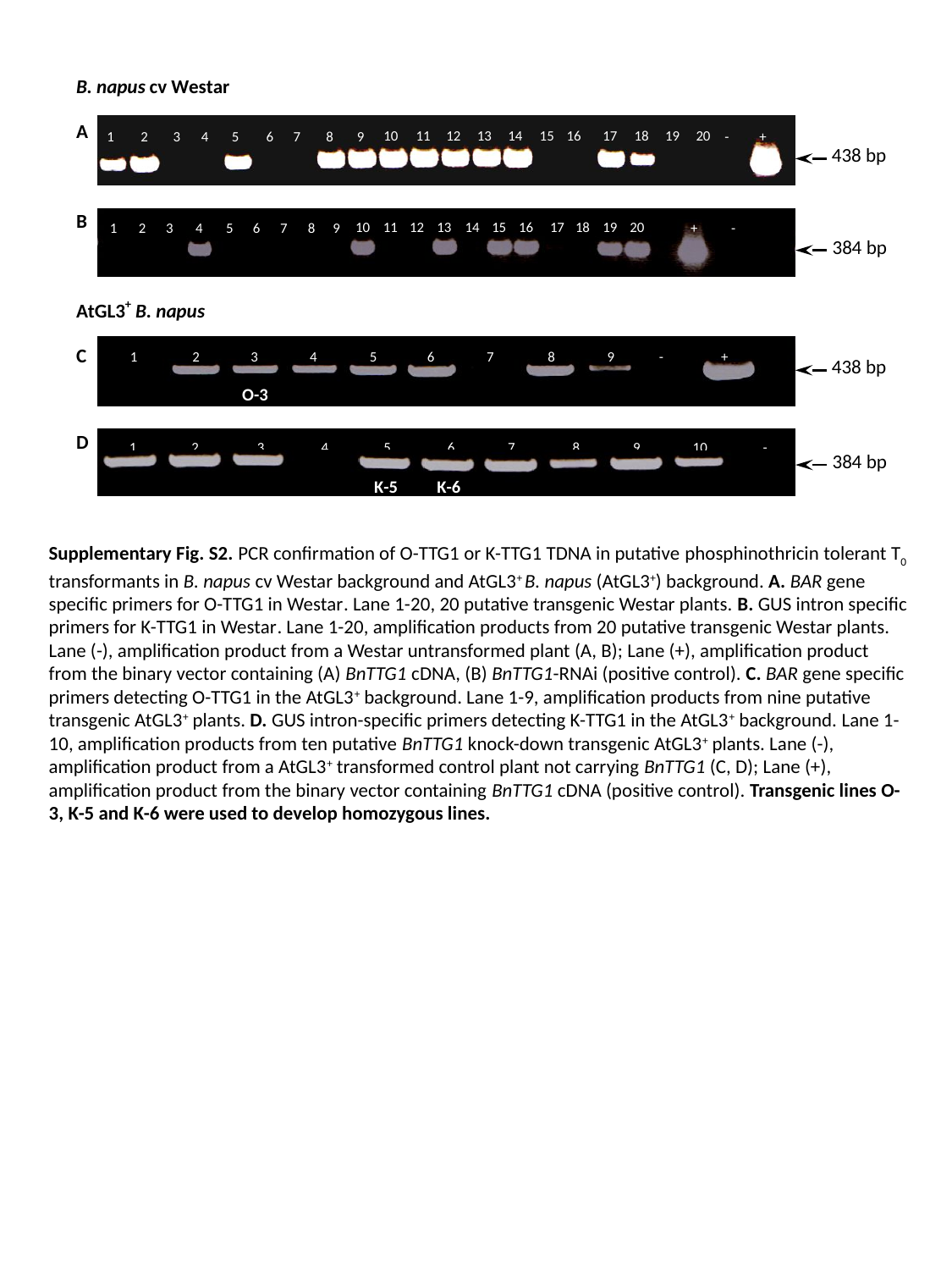

Supplementary Fig. S2. PCR confirmation of O-TTG1 or K-TTG1 TDNA in putative phosphinothricin tolerant T0 transformants in B. napus cv Westar background and AtGL3+ B. napus (AtGL3+) background. A. BAR gene specific primers for O-TTG1 in Westar. Lane 1-20, 20 putative transgenic Westar plants. B. GUS intron specific primers for K-TTG1 in Westar. Lane 1-20, amplification products from 20 putative transgenic Westar plants. Lane (-), amplification product from a Westar untransformed plant (A, B); Lane (+), amplification product from the binary vector containing (A) BnTTG1 cDNA, (B) BnTTG1-RNAi (positive control). C. BAR gene specific primers detecting O-TTG1 in the AtGL3+ background. Lane 1-9, amplification products from nine putative transgenic AtGL3+ plants. D. GUS intron-specific primers detecting K-TTG1 in the AtGL3+ background. Lane 1-10, amplification products from ten putative BnTTG1 knock-down transgenic AtGL3+ plants. Lane (-), amplification product from a AtGL3+ transformed control plant not carrying BnTTG1 (C, D); Lane (+), amplification product from the binary vector containing BnTTG1 cDNA (positive control). Transgenic lines O-3, K-5 and K-6 were used to develop homozygous lines.

## Slide 4
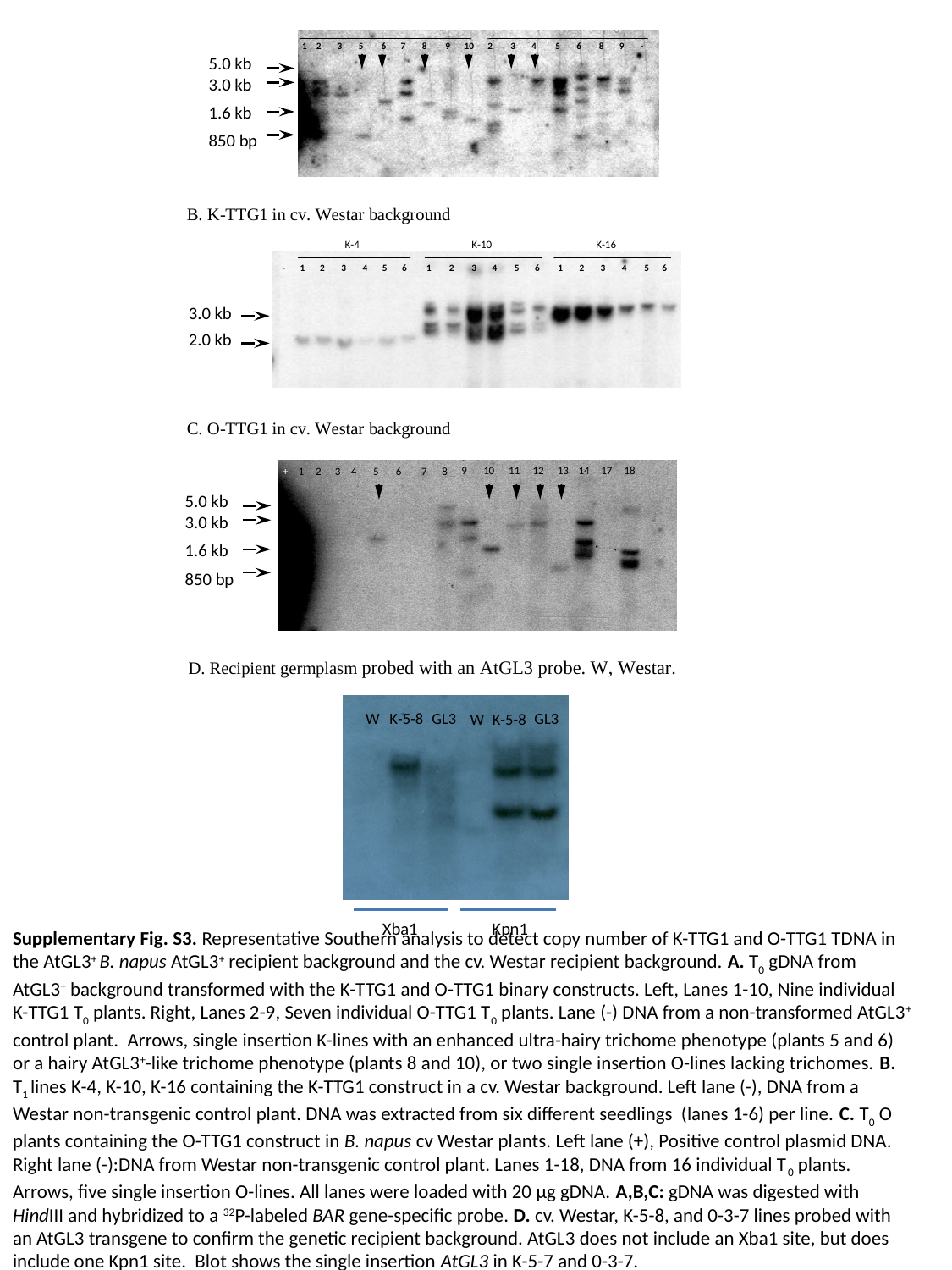

Supplementary Fig. S3. Representative Southern analysis to detect copy number of K-TTG1 and O-TTG1 TDNA in the AtGL3+ B. napus AtGL3+ recipient background and the cv. Westar recipient background. A. T0 gDNA from AtGL3+ background transformed with the K-TTG1 and O-TTG1 binary constructs. Left, Lanes 1-10, Nine individual K-TTG1 T0 plants. Right, Lanes 2-9, Seven individual O-TTG1 T0 plants. Lane (-) DNA from a non-transformed AtGL3+ control plant. Arrows, single insertion K-lines with an enhanced ultra-hairy trichome phenotype (plants 5 and 6) or a hairy AtGL3+-like trichome phenotype (plants 8 and 10), or two single insertion O-lines lacking trichomes. B. T1 lines K-4, K-10, K-16 containing the K-TTG1 construct in a cv. Westar background. Left lane (-), DNA from a Westar non-transgenic control plant. DNA was extracted from six different seedlings (lanes 1-6) per line. C. T0 O plants containing the O-TTG1 construct in B. napus cv Westar plants. Left lane (+), Positive control plasmid DNA. Right lane (-):DNA from Westar non-transgenic control plant. Lanes 1-18, DNA from 16 individual T0 plants. Arrows, five single insertion O-lines. All lanes were loaded with 20 μg gDNA. A,B,C: gDNA was digested with HindIII and hybridized to a 32P-labeled BAR gene-specific probe. D. cv. Westar, K-5-8, and 0-3-7 lines probed with an AtGL3 transgene to confirm the genetic recipient background. AtGL3 does not include an Xba1 site, but does include one Kpn1 site. Blot shows the single insertion AtGL3 in K-5-7 and 0-3-7.

## Slide 5
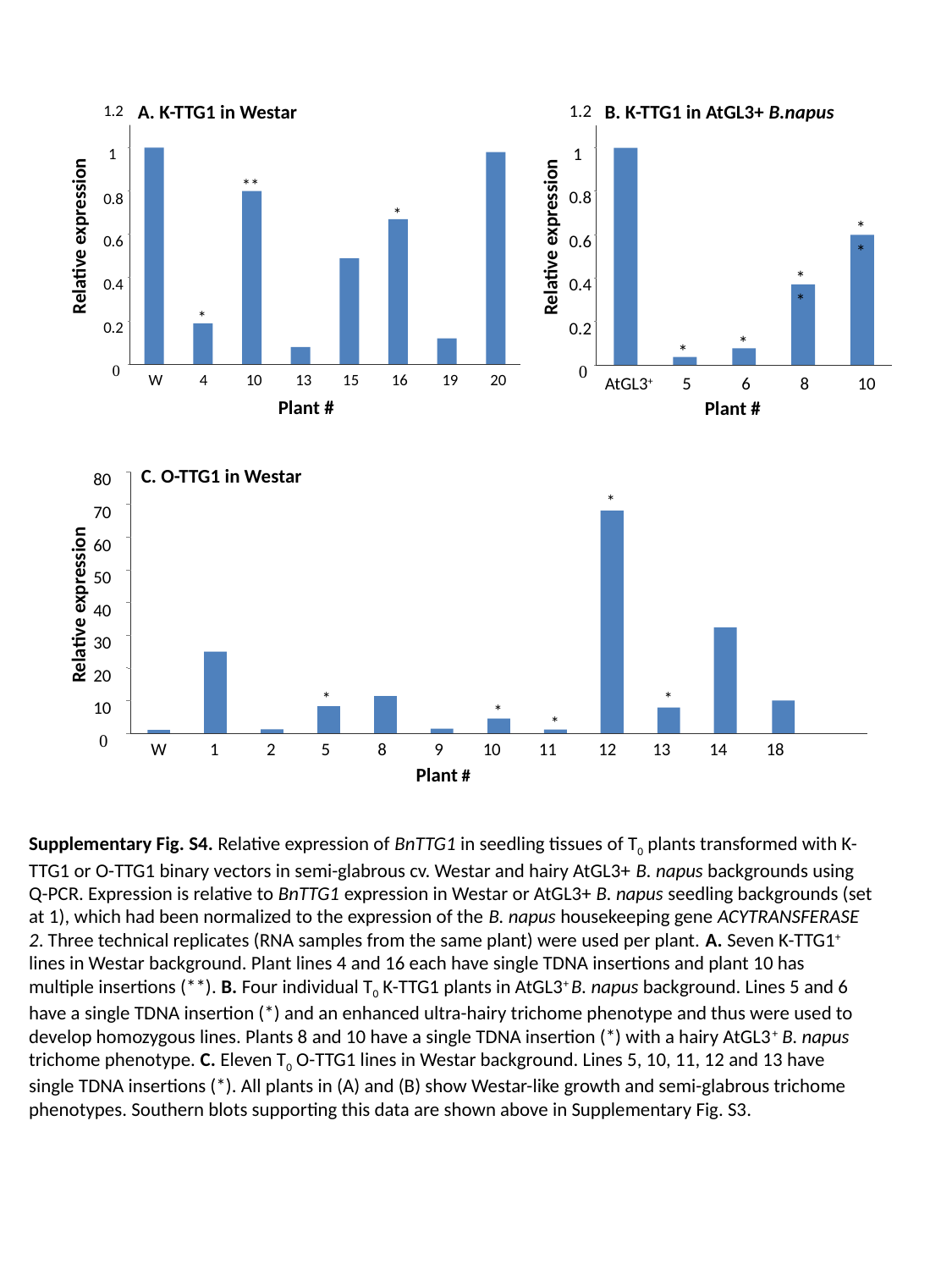

B. K-TTG1 in AtGL3+ B.napus
1.2
1
0.8
**
Relative expression
0.6
**
0.4
0.2
*
*
0
AtGL3+
5
6
8
10
Plant #
A. K-TTG1 in Westar
1.2
1
**
0.8
*
Relative expression
0.6
0.4
*
0.2
0
W
4
10
13
15
16
19
20
Plant #
C. O-TTG1 in Westar
80
*
70
60
50
Relative expression
40
30
20
*
*
*
10
*
0
W
1
2
5
8
9
10
11
12
13
14
18
Plant #
Supplementary Fig. S4. Relative expression of BnTTG1 in seedling tissues of T0 plants transformed with K-TTG1 or O-TTG1 binary vectors in semi-glabrous cv. Westar and hairy AtGL3+ B. napus backgrounds using Q-PCR. Expression is relative to BnTTG1 expression in Westar or AtGL3+ B. napus seedling backgrounds (set at 1), which had been normalized to the expression of the B. napus housekeeping gene ACYTRANSFERASE 2. Three technical replicates (RNA samples from the same plant) were used per plant. A. Seven K-TTG1+ lines in Westar background. Plant lines 4 and 16 each have single TDNA insertions and plant 10 has multiple insertions (**). B. Four individual T0 K-TTG1 plants in AtGL3+ B. napus background. Lines 5 and 6 have a single TDNA insertion (*) and an enhanced ultra-hairy trichome phenotype and thus were used to develop homozygous lines. Plants 8 and 10 have a single TDNA insertion (*) with a hairy AtGL3+ B. napus trichome phenotype. C. Eleven T0 O-TTG1 lines in Westar background. Lines 5, 10, 11, 12 and 13 have single TDNA insertions (*). All plants in (A) and (B) show Westar-like growth and semi-glabrous trichome phenotypes. Southern blots supporting this data are shown above in Supplementary Fig. S3.

## Slide 6
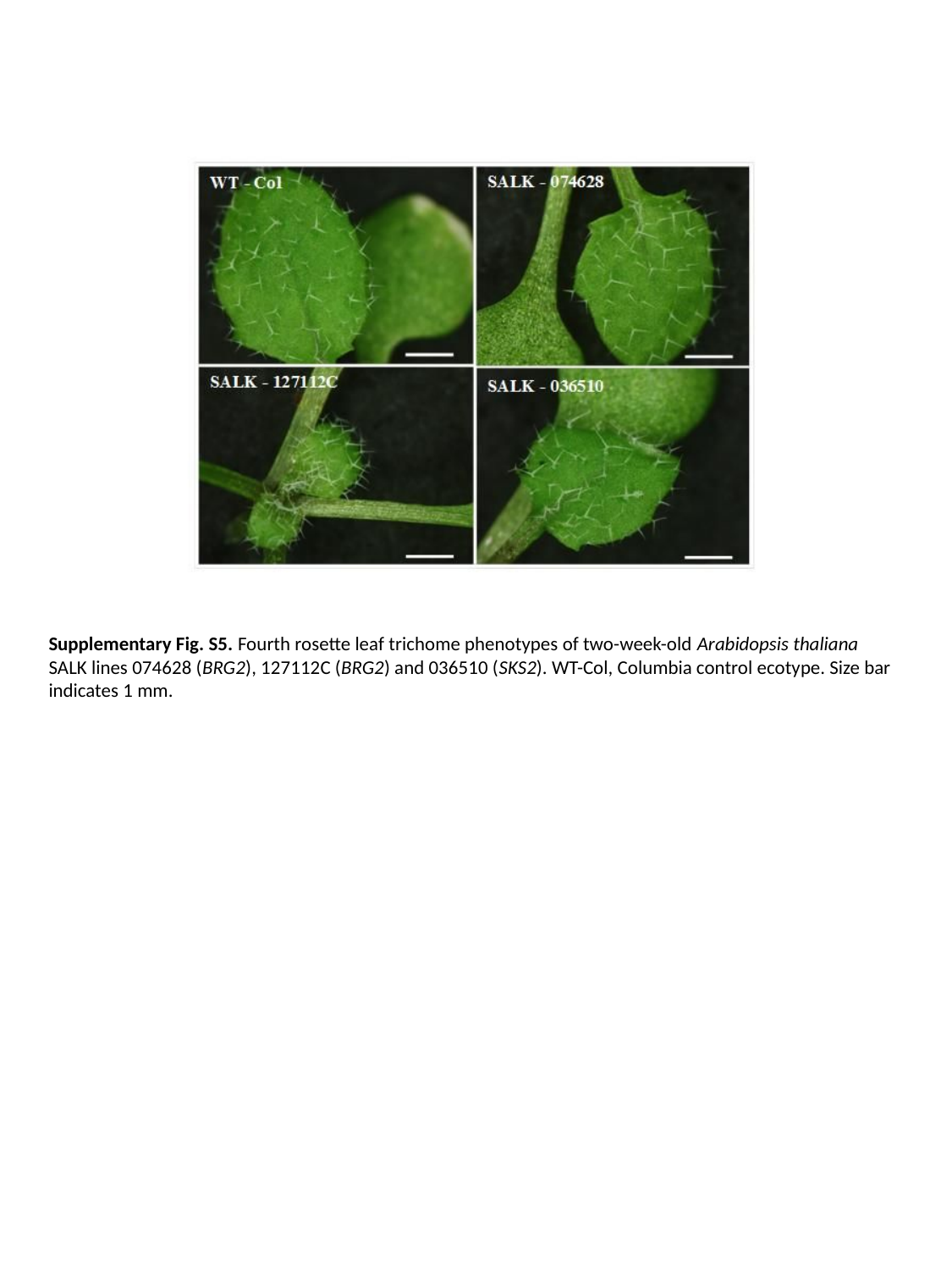

Supplementary Fig. S5. Fourth rosette leaf trichome phenotypes of two-week-old Arabidopsis thaliana SALK lines 074628 (BRG2), 127112C (BRG2) and 036510 (SKS2). WT-Col, Columbia control ecotype. Size bar indicates 1 mm.

## Slide 7
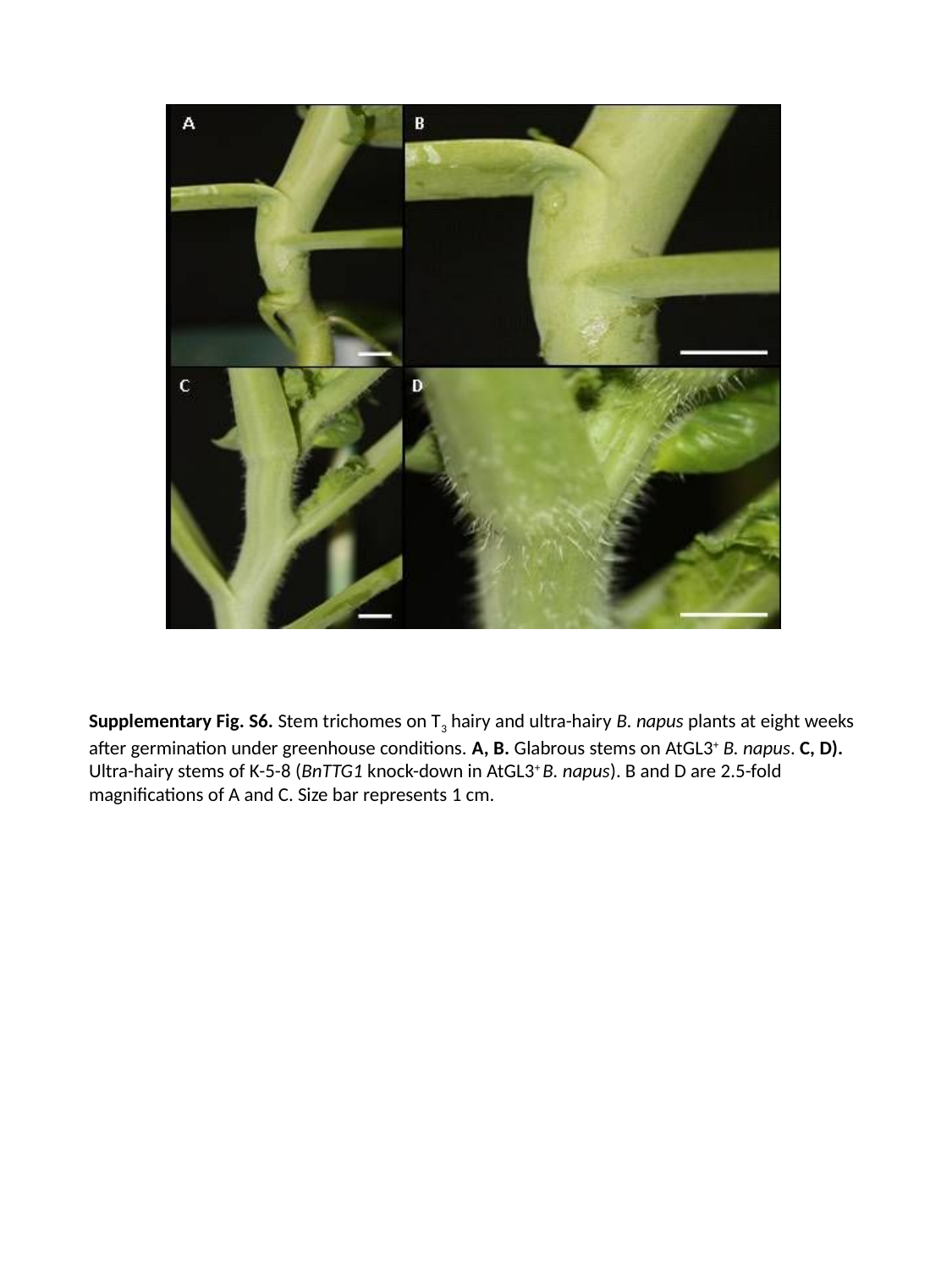

Supplementary Fig. S6. Stem trichomes on T3 hairy and ultra-hairy B. napus plants at eight weeks after germination under greenhouse conditions. A, B. Glabrous stems on AtGL3+ B. napus. C, D). Ultra-hairy stems of K-5-8 (BnTTG1 knock-down in AtGL3+ B. napus). B and D are 2.5-fold magnifications of A and C. Size bar represents 1 cm.

## Slide 8
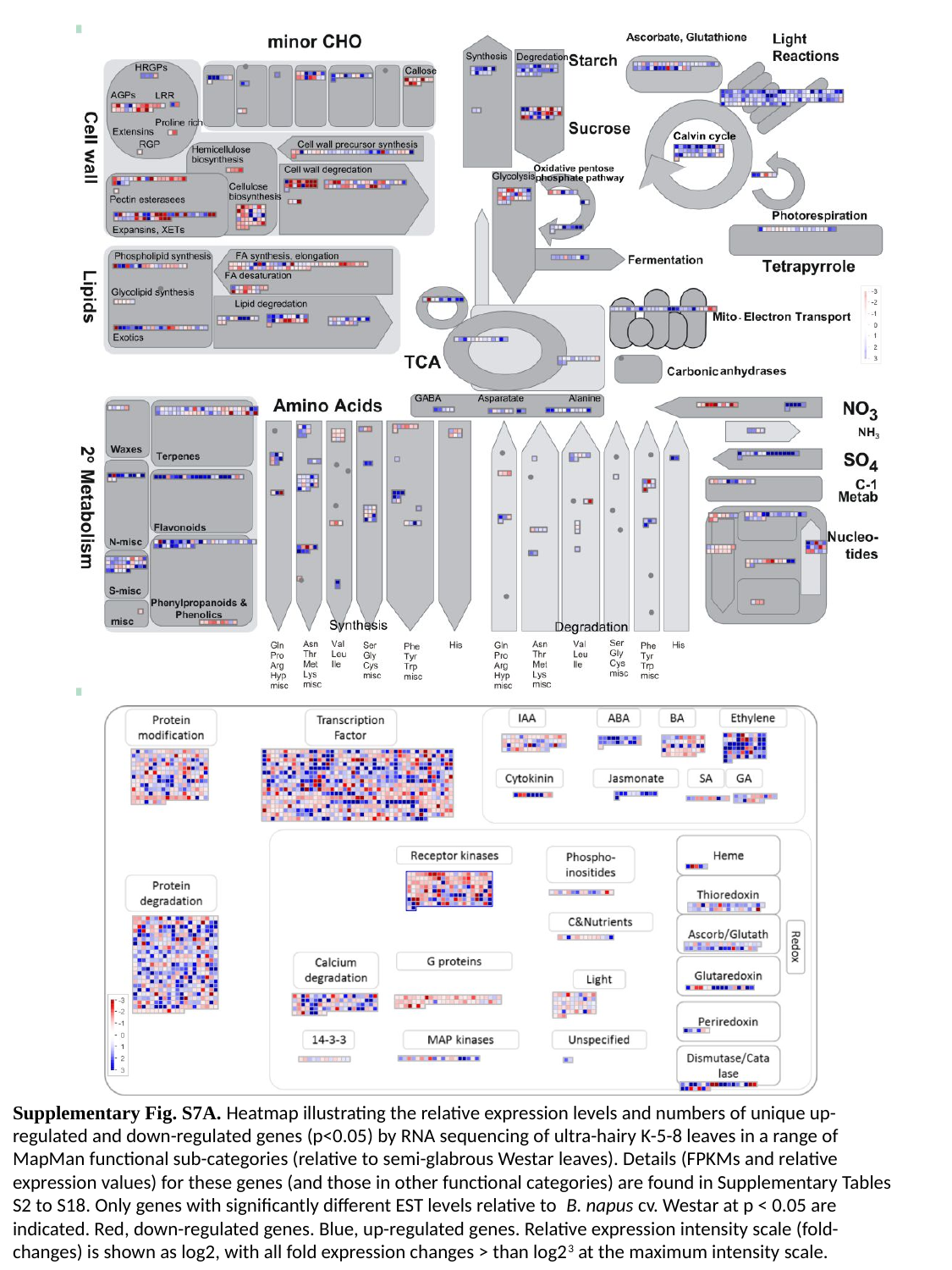

Supplementary Fig. S7A. Heatmap illustrating the relative expression levels and numbers of unique up-regulated and down-regulated genes (p<0.05) by RNA sequencing of ultra-hairy K-5-8 leaves in a range of MapMan functional sub-categories (relative to semi-glabrous Westar leaves). Details (FPKMs and relative expression values) for these genes (and those in other functional categories) are found in Supplementary Tables S2 to S18. Only genes with significantly different EST levels relative to B. napus cv. Westar at p < 0.05 are indicated. Red, down-regulated genes. Blue, up-regulated genes. Relative expression intensity scale (fold-changes) is shown as log2, with all fold expression changes > than log23 at the maximum intensity scale.

## Slide 9
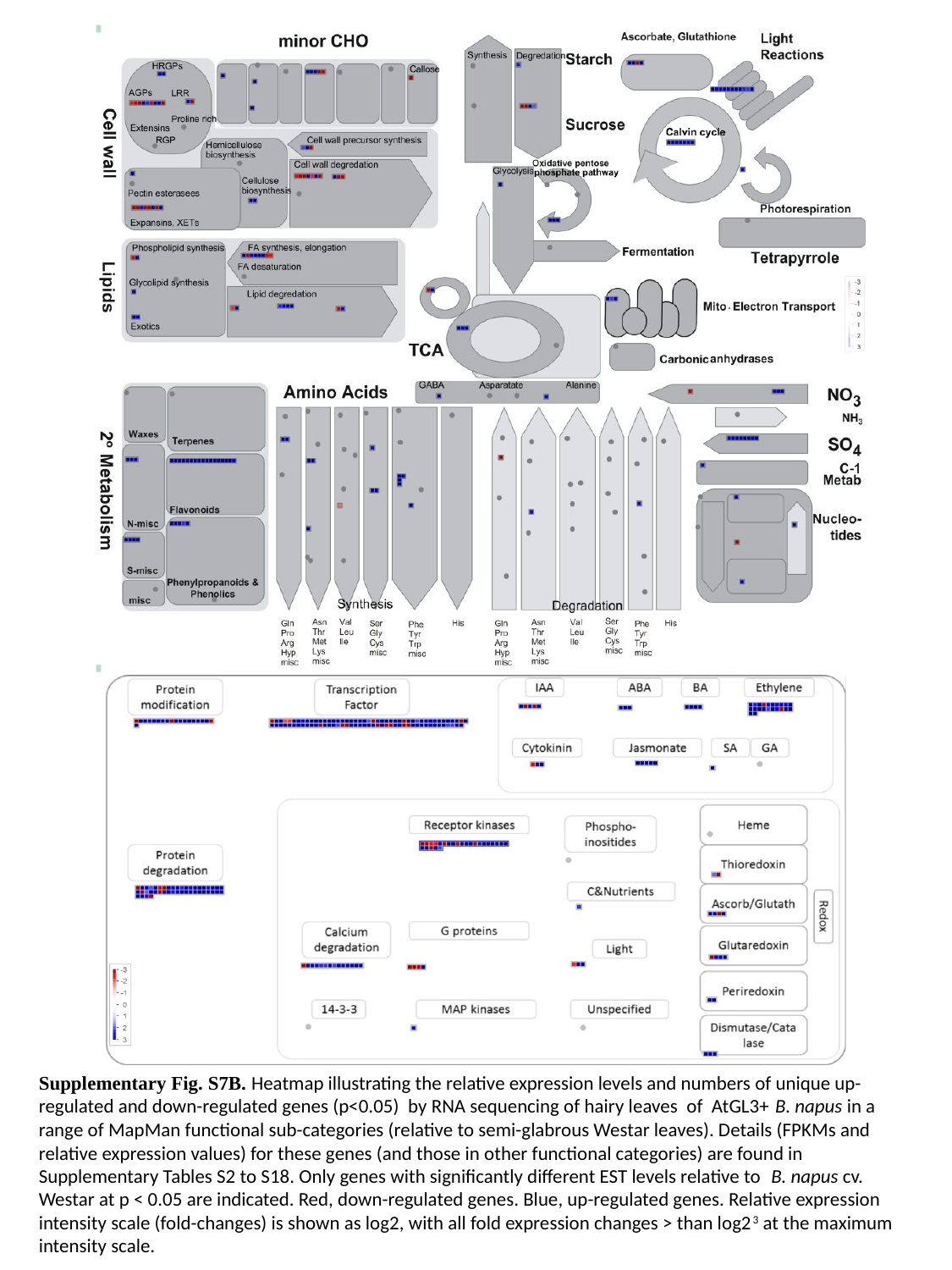

Supplementary Fig. S7B. Heatmap illustrating the relative expression levels and numbers of unique up-regulated and down-regulated genes (p<0.05) by RNA sequencing of hairy leaves of AtGL3+ B. napus in a range of MapMan functional sub-categories (relative to semi-glabrous Westar leaves). Details (FPKMs and relative expression values) for these genes (and those in other functional categories) are found in Supplementary Tables S2 to S18. Only genes with significantly different EST levels relative to B. napus cv. Westar at p < 0.05 are indicated. Red, down-regulated genes. Blue, up-regulated genes. Relative expression intensity scale (fold-changes) is shown as log2, with all fold expression changes > than log23 at the maximum intensity scale.

## Slide 10
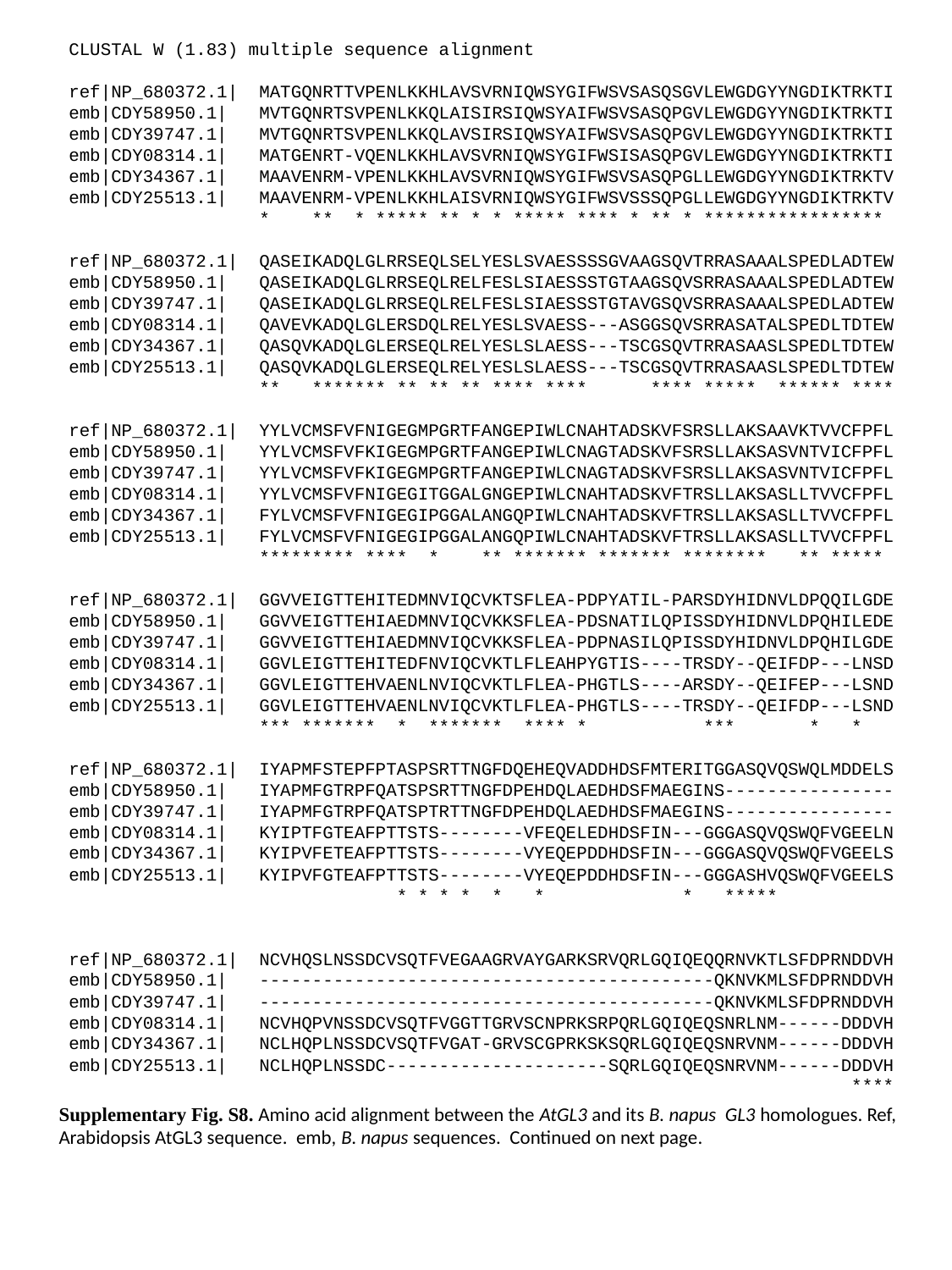

CLUSTAL W (1.83) multiple sequence alignment
ref|NP_680372.1| MATGQNRTTVPENLKKHLAVSVRNIQWSYGIFWSVSASQSGVLEWGDGYYNGDIKTRKTI
emb|CDY58950.1| MVTGQNRTSVPENLKKQLAISIRSIQWSYAIFWSVSASQPGVLEWGDGYYNGDIKTRKTI
emb|CDY39747.1| MVTGQNRTSVPENLKKQLAVSIRSIQWSYAIFWSVSASQPGVLEWGDGYYNGDIKTRKTI
emb|CDY08314.1| MATGENRT-VQENLKKHLAVSVRNIQWSYGIFWSISASQPGVLEWGDGYYNGDIKTRKTI
emb|CDY34367.1| MAAVENRM-VPENLKKHLAVSVRNIQWSYGIFWSVSASQPGLLEWGDGYYNGDIKTRKTV
emb|CDY25513.1| MAAVENRM-VPENLKKHLAISVRNIQWSYGIFWSVSSSQPGLLEWGDGYYNGDIKTRKTV
 * ** * ***** ** * * ***** **** * ** * *****************
ref|NP_680372.1| QASEIKADQLGLRRSEQLSELYESLSVAESSSSGVAAGSQVTRRASAAALSPEDLADTEW
emb|CDY58950.1| QASEIKADQLGLRRSEQLRELFESLSIAESSSTGTAAGSQVSRRASAAALSPEDLADTEW
emb|CDY39747.1| QASEIKADQLGLRRSEQLRELFESLSIAESSSTGTAVGSQVSRRASAAALSPEDLADTEW
emb|CDY08314.1| QAVEVKADQLGLERSDQLRELYESLSVAESS---ASGGSQVSRRASATALSPEDLTDTEW
emb|CDY34367.1| QASQVKADQLGLERSEQLRELYESLSLAESS---TSCGSQVTRRASAASLSPEDLTDTEW
emb|CDY25513.1| QASQVKADQLGLERSEQLRELYESLSLAESS---TSCGSQVTRRASAASLSPEDLTDTEW
 ** ******* ** ** ** **** **** **** ***** ****** ****
ref|NP_680372.1| YYLVCMSFVFNIGEGMPGRTFANGEPIWLCNAHTADSKVFSRSLLAKSAAVKTVVCFPFL
emb|CDY58950.1| YYLVCMSFVFKIGEGMPGRTFANGEPIWLCNAGTADSKVFSRSLLAKSASVNTVICFPFL
emb|CDY39747.1| YYLVCMSFVFKIGEGMPGRTFANGEPIWLCNAGTADSKVFSRSLLAKSASVNTVICFPFL
emb|CDY08314.1| YYLVCMSFVFNIGEGITGGALGNGEPIWLCNAHTADSKVFTRSLLAKSASLLTVVCFPFL
emb|CDY34367.1| FYLVCMSFVFNIGEGIPGGALANGQPIWLCNAHTADSKVFTRSLLAKSASLLTVVCFPFL
emb|CDY25513.1| FYLVCMSFVFNIGEGIPGGALANGQPIWLCNAHTADSKVFTRSLLAKSASLLTVVCFPFL
 ********* **** * ** ******* ******* ******** ** *****
ref|NP_680372.1| GGVVEIGTTEHITEDMNVIQCVKTSFLEA-PDPYATIL-PARSDYHIDNVLDPQQILGDE
emb|CDY58950.1| GGVVEIGTTEHIAEDMNVIQCVKKSFLEA-PDSNATILQPISSDYHIDNVLDPQHILEDE
emb|CDY39747.1| GGVVEIGTTEHIAEDMNVIQCVKKSFLEA-PDPNASILQPISSDYHIDNVLDPQHILGDE
emb|CDY08314.1| GGVLEIGTTEHITEDFNVIQCVKTLFLEAHPYGTIS----TRSDY--QEIFDP---LNSD
emb|CDY34367.1| GGVLEIGTTEHVAENLNVIQCVKTLFLEA-PHGTLS----ARSDY--QEIFEP---LSND
emb|CDY25513.1| GGVLEIGTTEHVAENLNVIQCVKTLFLEA-PHGTLS----TRSDY--QEIFDP---LSND
 *** ******* * ******* **** * *** * *
ref|NP_680372.1| IYAPMFSTEPFPTASPSRTTNGFDQEHEQVADDHDSFMTERITGGASQVQSWQLMDDELS
emb|CDY58950.1| IYAPMFGTRPFQATSPSRTTNGFDPEHDQLAEDHDSFMAEGINS----------------
emb|CDY39747.1| IYAPMFGTRPFQATSPTRTTNGFDPEHDQLAEDHDSFMAEGINS----------------
emb|CDY08314.1| KYIPTFGTEAFPTTSTS--------VFEQELEDHDSFIN---GGGASQVQSWQFVGEELN
emb|CDY34367.1| KYIPVFETEAFPTTSTS--------VYEQEPDDHDSFIN---GGGASQVQSWQFVGEELS
emb|CDY25513.1| KYIPVFGTEAFPTTSTS--------VYEQEPDDHDSFIN---GGGASHVQSWQFVGEELS
 * * * * * * * *****
ref|NP_680372.1| NCVHQSLNSSDCVSQTFVEGAAGRVAYGARKSRVQRLGQIQEQQRNVKTLSFDPRNDDVH
emb|CDY58950.1| -------------------------------------------QKNVKMLSFDPRNDDVH
emb|CDY39747.1| -------------------------------------------QKNVKMLSFDPRNDDVH
emb|CDY08314.1| NCVHQPVNSSDCVSQTFVGGTTGRVSCNPRKSRPQRLGQIQEQSNRLNM------DDDVH
emb|CDY34367.1| NCLHQPLNSSDCVSQTFVGAT-GRVSCGPRKSKSQRLGQIQEQSNRVNM------DDDVH
emb|CDY25513.1| NCLHQPLNSSDC---------------------SQRLGQIQEQSNRVNM------DDDVH
 ****
Supplementary Fig. S8. Amino acid alignment between the AtGL3 and its B. napus GL3 homologues. Ref, Arabidopsis AtGL3 sequence. emb, B. napus sequences. Continued on next page.
 n

## Slide 11
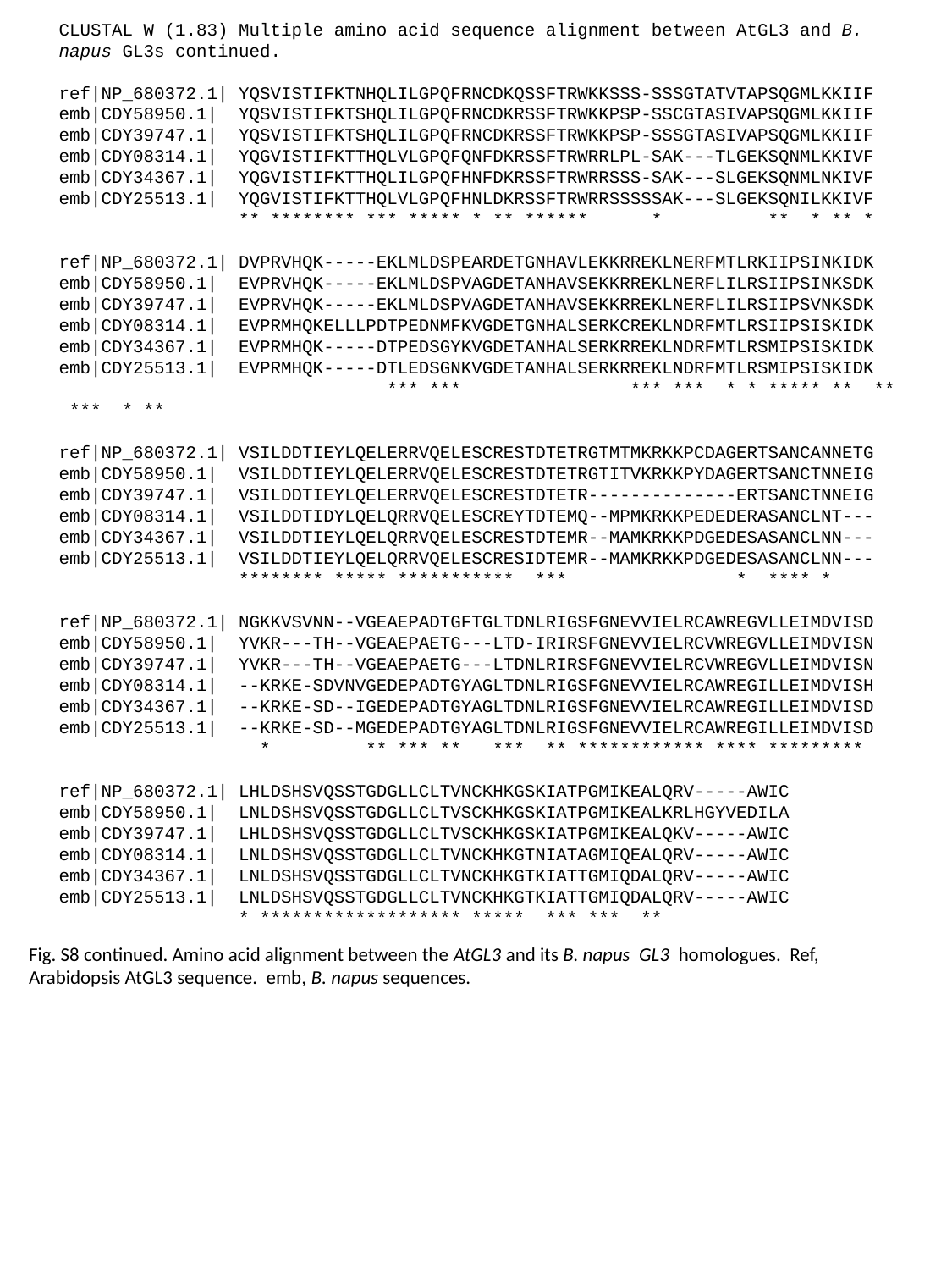

CLUSTAL W (1.83) Multiple amino acid sequence alignment between AtGL3 and B. napus GL3s continued.
ref|NP_680372.1| YQSVISTIFKTNHQLILGPQFRNCDKQSSFTRWKKSSS-SSSGTATVTAPSQGMLKKIIF
emb|CDY58950.1| YQSVISTIFKTSHQLILGPQFRNCDKRSSFTRWKKPSP-SSCGTASIVAPSQGMLKKIIF
emb|CDY39747.1| YQSVISTIFKTSHQLILGPQFRNCDKRSSFTRWKKPSP-SSSGTASIVAPSQGMLKKIIF
emb|CDY08314.1| YQGVISTIFKTTHQLVLGPQFQNFDKRSSFTRWRRLPL-SAK---TLGEKSQNMLKKIVF
emb|CDY34367.1| YQGVISTIFKTTHQLILGPQFHNFDKRSSFTRWRRSSS-SAK---SLGEKSQNMLNKIVF
emb|CDY25513.1| YQGVISTIFKTTHQLVLGPQFHNLDKRSSFTRWRRSSSSSAK---SLGEKSQNILKKIVF
 ** ******** *** ***** * ** ****** * ** * ** *
ref|NP_680372.1| DVPRVHQK-----EKLMLDSPEARDETGNHAVLEKKRREKLNERFMTLRKIIPSINKIDK
emb|CDY58950.1| EVPRVHQK-----EKLMLDSPVAGDETANHAVSEKKRREKLNERFLILRSIIPSINKSDK
emb|CDY39747.1| EVPRVHQK-----EKLMLDSPVAGDETANHAVSEKKRREKLNERFLILRSIIPSVNKSDK
emb|CDY08314.1| EVPRMHQKELLLPDTPEDNMFKVGDETGNHALSERKCREKLNDRFMTLRSIIPSISKIDK
emb|CDY34367.1| EVPRMHQK-----DTPEDSGYKVGDETANHALSERKRREKLNDRFMTLRSMIPSISKIDK
emb|CDY25513.1| EVPRMHQK-----DTLEDSGNKVGDETANHALSERKRREKLNDRFMTLRSMIPSISKIDK
 *** *** *** *** * * ***** ** ** *** * **
ref|NP_680372.1| VSILDDTIEYLQELERRVQELESCRESTDTETRGTMTMKRKKPCDAGERTSANCANNETG
emb|CDY58950.1| VSILDDTIEYLQELERRVQELESCRESTDTETRGTITVKRKKPYDAGERTSANCTNNEIG
emb|CDY39747.1| VSILDDTIEYLQELERRVQELESCRESTDTETR--------------ERTSANCTNNEIG
emb|CDY08314.1| VSILDDTIDYLQELQRRVQELESCREYTDTEMQ--MPMKRKKPEDEDERASANCLNT---
emb|CDY34367.1| VSILDDTIEYLQELQRRVQELESCRESTDTEMR--MAMKRKKPDGEDESASANCLNN---
emb|CDY25513.1| VSILDDTIEYLQELQRRVQELESCRESIDTEMR--MAMKRKKPDGEDESASANCLNN---
 ******** ***** *********** *** * **** *
ref|NP_680372.1| NGKKVSVNN--VGEAEPADTGFTGLTDNLRIGSFGNEVVIELRCAWREGVLLEIMDVISD
emb|CDY58950.1| YVKR---TH--VGEAEPAETG---LTD-IRIRSFGNEVVIELRCVWREGVLLEIMDVISN
emb|CDY39747.1| YVKR---TH--VGEAEPAETG---LTDNLRIRSFGNEVVIELRCVWREGVLLEIMDVISN
emb|CDY08314.1| --KRKE-SDVNVGEDEPADTGYAGLTDNLRIGSFGNEVVIELRCAWREGILLEIMDVISH
emb|CDY34367.1| --KRKE-SD--IGEDEPADTGYAGLTDNLRIGSFGNEVVIELRCAWREGILLEIMDVISD
emb|CDY25513.1| --KRKE-SD--MGEDEPADTGYAGLTDNLRIGSFGNEVVIELRCAWREGILLEIMDVISD
 * ** *** ** *** ** ************ **** *********
ref|NP_680372.1| LHLDSHSVQSSTGDGLLCLTVNCKHKGSKIATPGMIKEALQRV-----AWIC
emb|CDY58950.1| LNLDSHSVQSSTGDGLLCLTVSCKHKGSKIATPGMIKEALKRLHGYVEDILA
emb|CDY39747.1| LHLDSHSVQSSTGDGLLCLTVSCKHKGSKIATPGMIKEALQKV-----AWIC
emb|CDY08314.1| LNLDSHSVQSSTGDGLLCLTVNCKHKGTNIATAGMIQEALQRV-----AWIC
emb|CDY34367.1| LNLDSHSVQSSTGDGLLCLTVNCKHKGTKIATTGMIQDALQRV-----AWIC
emb|CDY25513.1| LNLDSHSVQSSTGDGLLCLTVNCKHKGTKIATTGMIQDALQRV-----AWIC
 * ******************* ***** *** *** **
Fig. S8 continued. Amino acid alignment between the AtGL3 and its B. napus GL3 homologues. Ref, Arabidopsis AtGL3 sequence. emb, B. napus sequences.

## Slide 12
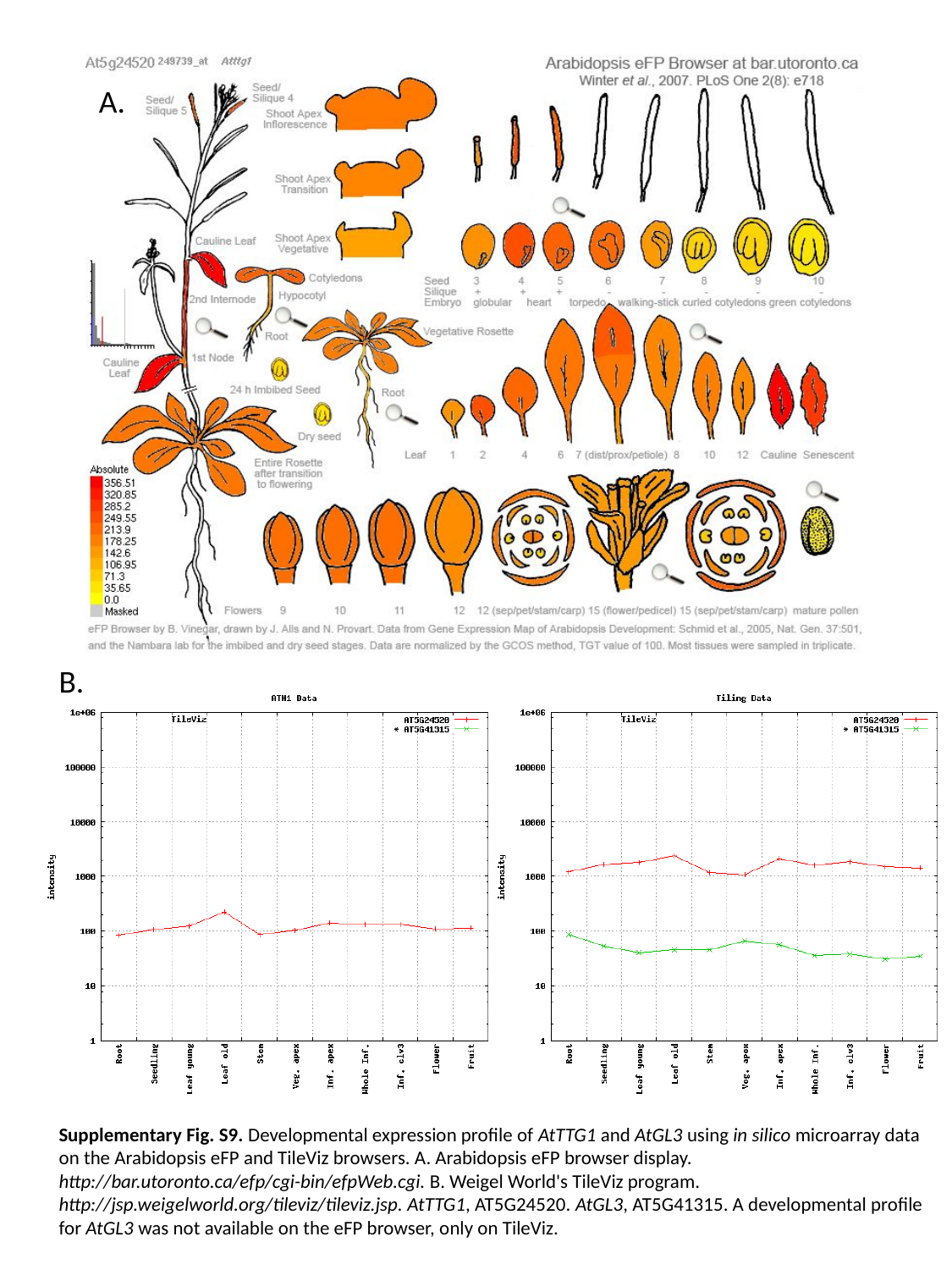

A.
B.
Supplementary Fig. S9. Developmental expression profile of AtTTG1 and AtGL3 using in silico microarray data on the Arabidopsis eFP and TileViz browsers. A. Arabidopsis eFP browser display. http://bar.utoronto.ca/efp/cgi-bin/efpWeb.cgi. B. Weigel World's TileViz program. http://jsp.weigelworld.org/tileviz/tileviz.jsp. AtTTG1, AT5G24520. AtGL3, AT5G41315. A developmental profile for AtGL3 was not available on the eFP browser, only on TileViz.
